# Multi-Omics Analysis Reveals the Protection of Gasdermin D in Concanavalin A-Induced Autoimmune Hepatitis

**DOI:** 10.1128/spectrum.01717-22

**Published:** 2022-08-16

**Authors:** Kaicen Wang, Wenrui Wu, Xianwan Jiang, Jiafeng Xia, Longxian Lv, Shengjie Li, Aoxiang Zhuge, Zhengjie Wu, Qiangqiang Wang, Shuting Wang, Lanjuan Li

**Affiliations:** a State Key Laboratory for Diagnosis and Treatment of Infectious Diseases, National Clinical Research Center for Infectious Diseases, Collaborative Innovation Center for Diagnosis and Treatment of Infectious Diseases, The First Affiliated Hospital, College of Medicine, Zhejiang University, Hangzhou, China; b Jinan Microecological Biomedicine Shandong Laboratory, Jinan, China; Shandong University

**Keywords:** gasdermin D, autoimmune hepatitis, intestinal barrier, gut microbiota, metabolome

## Abstract

Autoimmune hepatitis (AIH) is a progressive inflammation-associated liver injury. Pyroptosis is a novel inflammatory programmed cell death wherein gasdermin D (GSDMD) serves as the executioner. Our work challenged *Gsdmd*^−/−^ mice with concanavalin A (ConA) to try to unveil the actual role of GSDMD in AIH. After ConA injection, *Gsdmd*^−/−^ mice exhibited more severe liver damage characterized by a lower survival rate, more extensive hepatocyte necrosis and apoptosis, and higher serum transaminase levels, indicating the protection of GSDMD in ConA-induced AIH. Furthermore, the *Gsdmd*^−/−^ mice exhibited higher hepatic expression and serum levels of inflammatory cytokines (gamma interferon [IFN-γ], tumor necrosis factor alpha [TNF-α], and interleukin-17A [IL-17A]) and more infiltration of macrophages and neutrophils after ConA treatment than did wild-type (WT) mice. *Gsdmd*^−/−^ mice with AIH showed increased hepatic l-glutamine levels but decreased glycerophospholipid metabolites levels. L-glutamine levels showed positive correlations while glycerophospholipid metabolites showed negative associations with liver injury indexes and inflammation markers. We further observed a destroyed intestinal barrier in *Gsdmd*^−/−^ mice after ConA injection as indicated by decreased transcriptional expressions of *Tjp1*, *Ocln*, *Reg3g*, and *Muc2*. ConA-treated *Gsdmd*^−/−^ mice also exhibited higher serum LPS binding protein (LBP) concentrations and hepatic *Tlr4* and *Cd14* mRNA levels. Further fecal 16S rRNA gene sequencing demonstrated decreased relative abundances of *Lactobacillus* and *Roseburia* but increased relative abundances of *Allobaculum* and *Dubosiella* in *Gsdmd*^−/−^ mice with AIH. *Lactobacillus* was negatively correlated with liver injury and inflammation indexes and positively associated with *Ocln*, *Muc2*, and *Reg3g* levels. *Allobaculum* was positively related to liver injury and inflammatory cytokines and negatively correlated with gut barrier indexes.

**IMPORTANCE** Our study provides the first direct clues to the protective role of gasdermin D (GSDMD) in autoimmune hepatitis (AIH). We demonstrated that *Gsdmd* knockout exacerbated concanavalin A (ConA)-induced AIH in mice. It may be due to the destroyed intestinal barrier and changes in certain intestinal microbes and hepatic metabolites resulting in increased liver injury and inflammation in ConA-treated *Gsdmd*^−/−^ mice. This finding suggested a nonnegligible role of GSDMD in AIH and also confirmed its physiological nonpyroptosis effects on the host. The role of GSDMD in autoimmune liver diseases or other liver diseases is complex and intriguing, deserving deep investigation.

## INTRODUCTION

Pyroptosis is a new type of inflammatory programmed cell death, wherein gasdermin D (GSDMD) functions as the pivotal regulator and executioner (1, 2). Upon activation by lipopolysaccharide (LPS) and inflammasome, proinflammatory caspases (caspase 1 and caspase 11) cut off the linker between the GSDMD N- and C-terminal domains (2–5). Then, the freed GSDMD-N fragments translocate to the cytomembrane and form pores, resulting in cell lytic death and secretion of inflammatory cytokines such as interleukin-1β (IL-1β) and IL-18 (6). GSDMD-mediated pyroptosis plays an important role in the development of various diseases. It is required for lethal sepsis since mice lacking *Gsdmd* tolerated LPS-induced endotoxemia (7). It is worth noting that the role of GSDMD in different liver diseases is largely controversial and highly correlated with liver pathologies. On the one hand, the GSDMD-N domain was reported to have detrimental roles in metabolic liver diseases, such as nonalcoholic fatty liver disease (NAFLD) and nonalcoholic steatohepatitis (NASH) as removal of *Gsdmd* could protect mice from these kinds of liver diseases (8, 9). On the other hand, researchers have reported the hepatoprotective effect of GSDMD in hemorrhagic shock with resuscitation (HS/R) and acetaminophen (APAP) overdose-induced reactive oxygen species (ROS)-related liver injury as *Gsdmd*^−/−^ mice showed more severe liver damage (10).

Autoimmune hepatitis (AIH) is a progressive inflammatory disorder of unknown primary causes (11, 12). It is characterized by immune-mediated hepatocyte death and excessive inflammatory cytokine production (13). T cell-mediated hepatitis induced by concanavalin A (ConA) can mimic AIH in humans to some extent and is therefore considered a well-established experimental model of immune-mediated liver injury (14, 15). Previous studies have largely shown that apoptosis and necrosis are two main modes of liver cell death in AIH (16). The gut is the largest immune organ in the body (17). The collection of bacteria that inhabit the gut is termed the gut microbiota, and it acts as an “invisible organ” that exerts profound impacts on the host’s immunity (18). Various studies demonstrated the close relationship between gut microbiota and AIH. Germfree (GF) mice are resistant to ConA-induced liver injury (19). Manipulation of microorganisms in mice, via either antibiotics or probiotics, could substantially improve ConA-induced hepatocyte death and inflammation (20, 21).

However, there are few studies focused on the role of hepatocyte pyroptosis in AIH. Researchers have found that NLRP3, cleaved caspase 1, and IL-1β were upregulated in the liver after intravenous application of ConA and that blocking NLRP3, caspase 1, or IL-1β could dampen the development of liver damage (22). These provided preliminary evidence of the relationship between AIH, the inflammasome, and pyroptosis. Nevertheless, as GSDMD is the executor of pyroptosis, its direct effect on autoimmune liver injury and its potential relationship with the gut microbiota remained to be investigated.

In this study, globally *Gsdmd*-deficient mice were challenged with ConA to investigate the direct contribution of GSDMD to immune-driven hepatitis. We tried to elucidate the related mechanisms from the aspects of the intestinal barrier, gut microbiome, and fecal and hepatic metabolome.

## RESULTS

### *Gsdmd*^−/−^ mice showed more severe liver damage after ConA injection.

**FIG 1:**
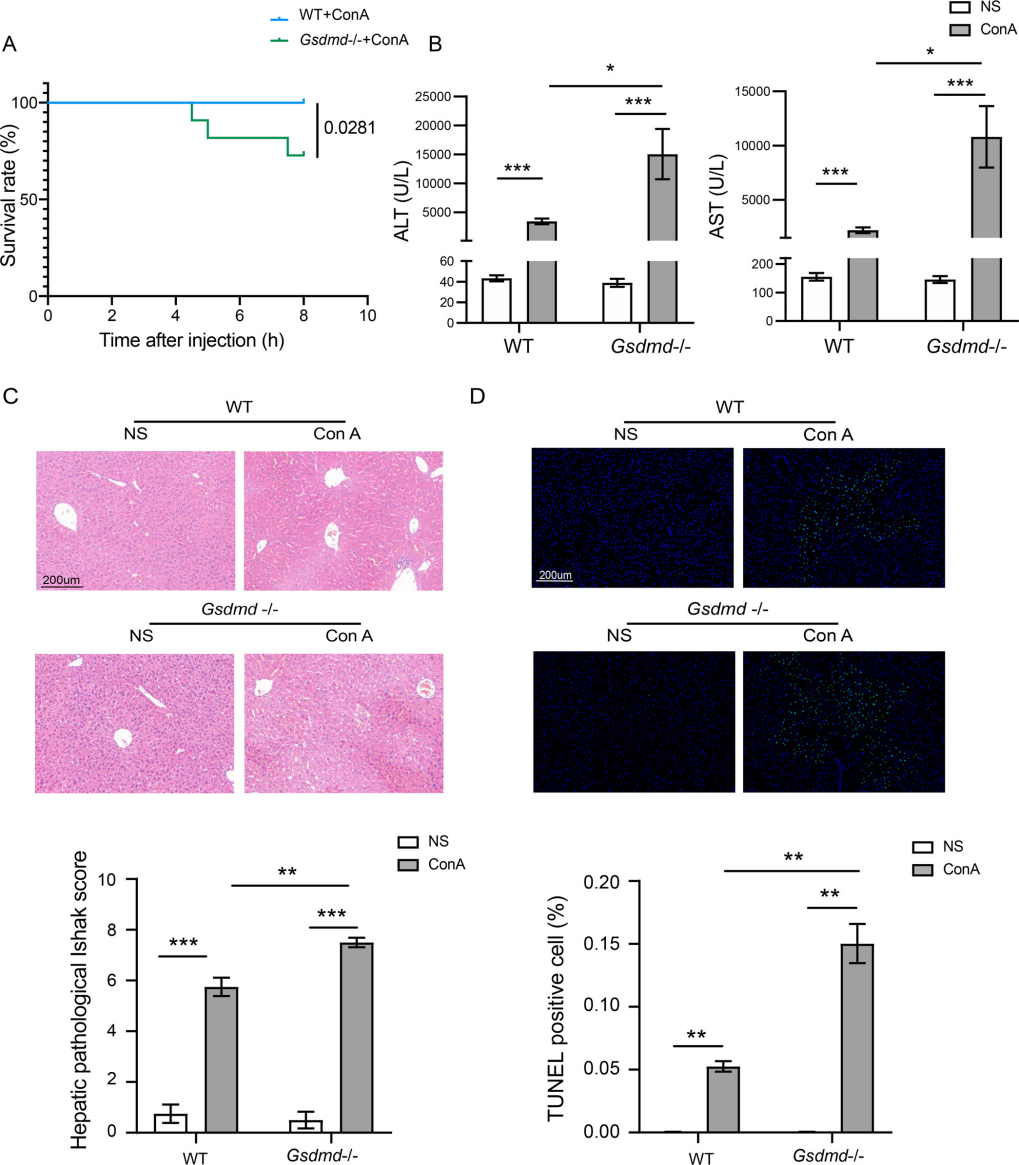
*Gsdmd*^−/−^ mice showed more severe liver damage after ConA injection. (A) Survival rate curve of mice 8 h after ConA injection in the WT+ConA and *Gsdmd*^−/−^+ConA groups; (B) ALT and AST levels in the four groups; (C) representative liver H&E staining images of the four groups and their corresponding hepatic pathological Ishak score (bar, 200 μm); (D) representative liver 4′,6-diamidino-2-phenylindole (DAPI)–TUNEL immunofluorescence staining and percentage of TUNEL-positive cells in each group (bar, 200 μm). The data are shown as the means ± SEM. *, *P* < 0.05; **, *P* < 0.01; ***, *P* < 0.001; ****, *P* < 0.0001.

Severe immune hepatitis in mice was established 8 h after injection of 15 mg of ConA per kg of body weight. As shown in the survival curve, *Gsdmd^−/−^* mice suffered higher mortality, with a 72.73% survival rate after the ConA challenge in the *Gsdmd*^−/−^+ConA group, whereas the wild-type (WT) mice all survived in the WT+ConA group (Fig. 1A). Alanine aminotransferase (ALT) and aspartate transaminase (AST) are considered the major indicators of liver injury. In contrast to the normal liver function in the WT+NS (normal saline) group, ConA treatment significantly increased the levels of these parameters in the WT+ConA group (Fig. 1B and see Table S1 in the supplemental material). *Gsdmd* depletion remarkably increased the elevation of ALT and AST levels in the *Gsdmd*^−/−^+ConA group compared to the WT+ConA group (Fig. 1B and Table S1). Furthermore, the Ishak score, which includes factors such as inflammation of the liver portal area and lobules and confluent necrosis of hepatocytes, was used to evaluate the liver pathological injury. As shown in the hematoxylin and eosin (H&E) staining of liver tissues, *Gsdmd*^−/−^ mice exhibited much more inflammation of the portal area and lobules as well as wider areas of liver confluent necrosis after ConA injection in the *Gsdmd*^−/−^+ConA group than the WT+ConA group (Fig. 1C). The significantly increased Ishak score further indicated the advanced liver damage in the *Gsdmd*^−/−^+ConA group (Fig. 1C). Immune-mediated hepatitis is always accompanied by apoptosis in addition to liver necrosis (23). Thus, we assessed the status of hepatocyte cell apoptosis with the TUNEL (terminal deoxynucleotidyltransferase-mediated dUTP-biotin nick end labeling) assay. The results showed that mice in the *Gsdmd*^−/−^+ConA group experienced more extensive apoptosis of hepatocytes than the WT+ConA group with about 3 times the percentage of TUNEL-positive cells (14.53% versus 5.42%) (Fig. 1D). Serological and pathological findings and TUNEL assay results all showed no notable changes in the WT+NS and *Gsdmd*^−/−^+NS groups.

Overall, *Gsdmd* knockout mice demonstrated a higher susceptibility to ConA-induced liver injury than the conventional ones.

### *Gsdmd*^−/−^ mice have more severe hepatic and systemic inflammation after ConA injection.

**FIG 2:**
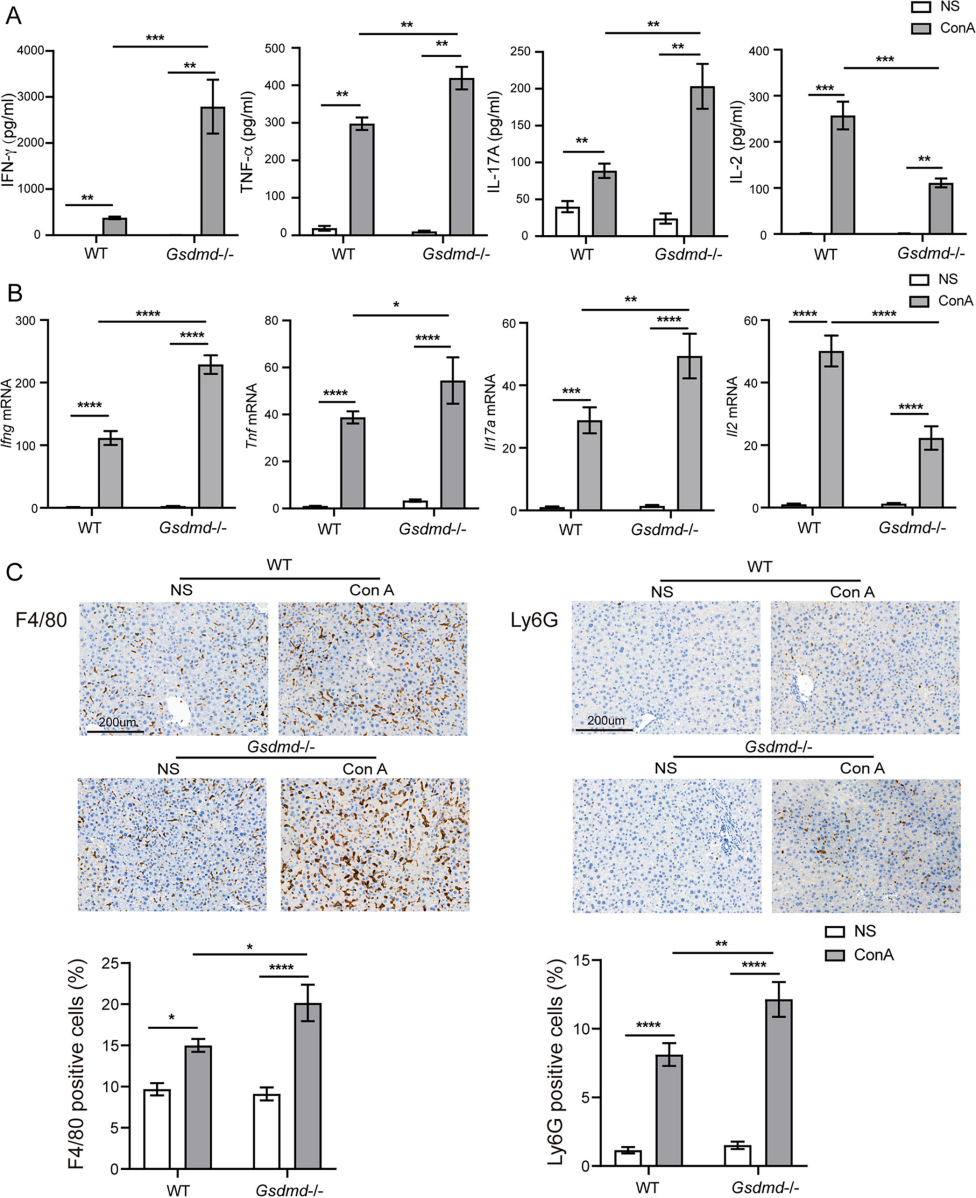
*Gsdmd*^−/−^ mice have more severe systemic and hepatic inflammation after ConA injection. (A) Serum levels of IFN-γ, TNF-α, IL-17A, and IL-2 in the four groups; (B) hepatic transcriptional levels of *Ifng*, *Tnf*, *Il17a*, and *Il2* in the four groups; (C) representative liver anti-F4/80 and anti-Ly6G immunohistochemical images of the four groups and percentages of F4/80- and Ly6G-positive cells, respectively (bar, 200 μm). The data are shown as the means ± SEM. *, *P* < 0.05; **, *P* < 0.01; ***, *P* < 0.001; ****, *P* < 0.0001.

We further assessed the systemic and hepatic inflammatory responses. The serum cytokine test showed that in contrast to the WT+NS group, ConA treatment elicited a cytokine storm in the WT+ConA group with dramatic increases in the levels of the detected cytokines (Table S2). Compared to the WT+ConA group, removing *Gsdmd* significantly increased circulating levels of chemokines (granulocyte-macrophage colony-stimulating factor [GM-CSF] and KC) and inflammatory cytokines (gamma interferon [IFN-γ], IL-1β, IL-3, IL-9, IL-13, IL-17A, and tumor necrosis factor alpha [TNF-α]) but decreased levels of IL-2, IL-5, IL-12(p40), and IL-12(p70) after ConA challenge in the *Gsdmd*^−/−^+ConA group (Fig. 2A and Table S2).

Consistently, the hepatic transcriptional results of some representative cytokines showed that compared to the WT+NS group, the expression levels of *Ifng*, *Tnf*, *Il1b*, *Il17a*, *Il2*, and *Il12a* increased dramatically in the WT+ConA group. The expressions of *Ifng*, *Il17a*, and *Tnf* were upregulated while the expression of *Il2* was downregulated in the *Gsdmd*^−/−^+ConA group in comparison to the WT+ConA group (Fig. 2B and Fig. S1A). Furthermore, immunohistochemical analysis of the F4/80 and Ly6G markers on macrophages and neutrophils in the liver suggested that compared to the WT+NS group, ConA injection triggered marked increases in the recruitment of hepatic macrophages and neutrophils as indicated by the higher number of F4/80- and Ly6G-positive cells in the WT+ConA group (Fig. 2C). In addition, it could be seen that macrophage and neutrophil infiltration in the livers of the *Gsdmd*^−/−^+ConA group was more significant than those in the WT+ConA group as illustrated by the significantly increased hepatic staining intensity of F4/80 and Ly6G (Fig. 2C).

These results collectively indicated that ConA challenge developed more severe systemic and hepatic inflammatory responses in *Gsdmd*^−/−^ mice than WT mice.

### Depletion of *Gsdmd* exacerbates intestinal barrier damage and LPS stimulation after ConA treatment.

**FIG 3:**
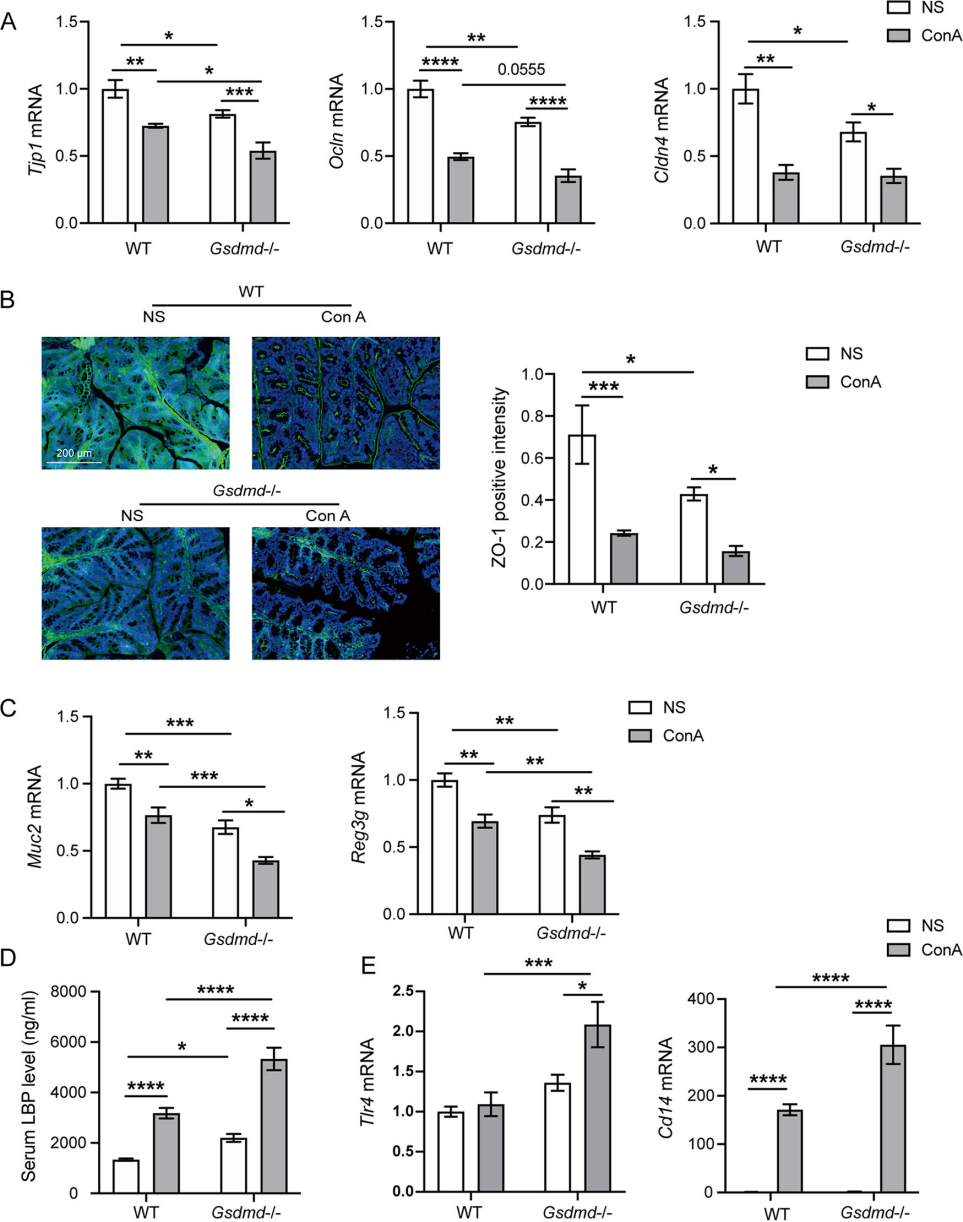
Depletion of *Gsdmd* exacerbated intestinal barrier damage and LPS stimulation after ConA treatment. (A) Colonic transcriptional expression of *Tjp1*, *Ocln*, and *Cldn4* in the four groups; (B) representative colon ZO-1 immunofluorescence staining images of the four groups and ZO-1 positive intensity (bar, 200 μm); (C) colon transcriptional expression of *Reg3g* and *Muc2* in the four groups; (D) serum level of LBP in the four groups; (E) hepatic transcriptional expression of *Tlr4* and *Cd14* in the four groups. The data are shown as the means ± SEM. *, *P* < 0.05; **, *P* < 0.01; ***, *P* < 0.001; ****, *P* < 0.0001.

Increased intestinal barrier permeability, also termed “the leaky gut,” played an important role in the occurrence and development of AIH through the gut-liver axis (21, 24). Intestinal tight junction (TJ) proteins are the fundamental basis of the intestinal barrier, and we detected the mRNA levels of TJ proteins such as zonula occludens protein-1 (ZO-1, encoded by *Tjp1*), occludin, and claudin 4 in the colon. The results showed that the expressions of *Tjp1*, *Ocln*, and *Cldn4* were downregulated in the *Gsdmd*^−/−^+NS group compared to the WT+NS group, indicating disruption of the intestinal barrier after *Gsdmd* knockout in the basement (Fig. 3A). ConA challenge remarkably impaired the integrity of the gut barrier with significantly decreased expression levels of *Tjp1*, *Ocln*, and *Cldn4* in the WT+ConA group compared to the WT+NS group (Fig. 3A). ConA injection induced a more remarkable inhibition of the expressions of *Tjp1* and *Ocln* in the *Gsdmd*^−/−^+ConA group than in the WT+ConA group (Fig. 3A). Consistently, further immunofluorescence staining of ZO-1 in the colon tissues also suggested increased TJ defects in the *Gsdmd*^−/−^+NS group compared to the WT+NS group as indicated by weaker intensity and lower density of the ZO-1 fluorescence (Fig. 3B). ConA injection resulted in a significant loss of ZO-1 in the WT+ConA group compared to the WT+NS group (Fig. 3B). Furthermore, ConA treatment caused a relatively low fluorescence intensity and density of ZO-1 in the *Gsdmd*^−/−^+ConA group compared to the WT+ConA group. These results indicated that aggravated liver damage in the *Gsdmd*^−/−^+ConA group was accompanied by more severe intestinal barrier damage (Fig. 3B).

Antimicrobial peptides and mucins are major components of the intestinal chemical barrier and play crucial roles in preventing bacterial translocation (25, 26). The mRNA expressions of antimicrobial peptides *Reg3g* and *Muc2* was further examined. Results showed that the expressions of *Reg3g* and *Muc2* were significantly downregulated in the *Gsdmd*^−/−^+NS group compared to the WT+NS group, reflecting a destroyed intestinal mucosal barrier caused by *Gsdmd* depletion (Fig. 3C). ConA injection suppressed the expressions of *Reg3g* and *Muc2* mRNA in the WT+ConA group compared to the WT+NS group (Fig. 3C). The *Gsdmd*^−/−^+ConA group displayed significant decreases in the expression of *Reg3g* and *Muc2* in contrast to the WT+ConA group, indicating a more severe mucosal barrier damage in *Gsdmd*^−/−^ mice with AIH (Fig. 3C).

When the intestinal barrier was impaired, the intestinal permeability was increased, and bacterial LPS could translocate to the liver through the portal circulation. LPS binding protein (LBP) is a stable index reflecting LPS exposure (27). Thus, we further quantified the serum LBP level in the four groups. The serum LBP level was higher in the *Gsdmd*^−/−^+NS group than in the WT+NS group (Fig. 3D). ConA-induced acute liver injury caused a significant increase in the concentration of LBP in the WT+ConA group compared to the WT+NS group (Fig. 3D). *Gsdmd*^−/−^ mice exhibited a remarkably elevated LBP level after ConA injection in the *Gsdmd*^−/−^+ConA group compared to the WT+ConA group (Fig. 3D).

Translocated LPS can activate the TLR4 receptor on the surface of hepatic Kupffer cells to elicit a hepatic immune response, trigger inflammatory cytokine release, and further promote liver damage. Thus, we next detected the hepatic mRNA expression levels of *Tlr4* and its coreceptor *Cd14* and found that their levels were slightly upregulated in the *Gsdmd*^−/−^+NS group compared to the WT+NS group (Fig. 3E). The expression of *Tlr4* was similar, whereas the expression of *Cd14* was dramatically stimulated after ConA injection in the WT+ConA group compared to the WT+NS group (Fig. 3E). In contrast to the WT+ConA group, ConA challenge significantly upregulated the expression level of *Tlr4* and *Cd14* in the *Gsdmd*^−/−^+ConA group (Fig. 3E).

In conclusion, *Gsdmd*^−/−^ mice showed increased intestinal barrier damage and hepatic LPS stimulation after ConA injection, which may contribute to the aggravation of *Gsdmd* deficiency to ConA-induced hepatitis.

### *Gsdmd* depletion reshaped and stabilized a distinct microbial structure in mice after ConA treatment.

Intestinal barrier function is closely associated with the composition and metabolic function of gut microbes. Thus, feces were collected to evaluate the changes in microbial structure in the four groups.

The *Gsdmd*^−/−^+NS group has a richer microbial community than the WT+NS group as indicated by increased indexes of observed species and Chao1 (Fig. S1B). However, after ConA treatment, the *Gsdmd*^−/−^+ConA group experienced reduced levels of observed species and Chao1 compared to the *Gsdmd*^−/−^+NS group (Fig. S1B). The levels of richness of the microbial community were similar between the WT+NS and WT+ConA groups, as well as between the WT+ConA and *Gsdmd*^−/−^+ConA groups (Fig. S1B). Values for Shannon, an index indicating the diversity of the microbial community, were similar between the four groups (Fig. S1B).

**FIG 4:**
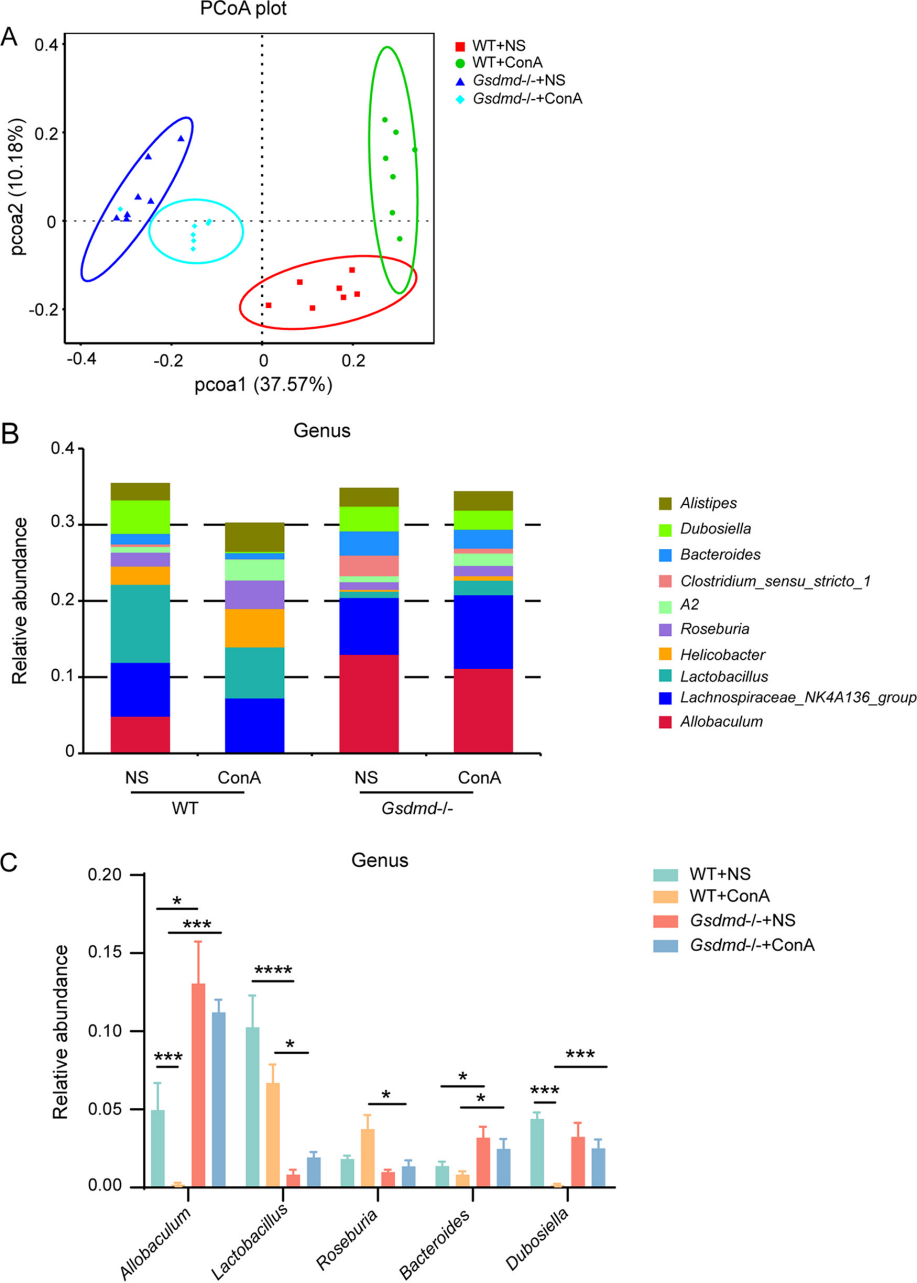
*Gsdmd* depletion reshaped a distinctive microbial structure and reduced microbial dysbiosis in mice after ConA treatment. (A) PCoA plot between the WT+NS, WT+ConA, *Gsdmd*^−/−^+NS, and *Gsdmd*^−/−^+ConA groups; (B) histogram of the top 10 most abundant genera in the four groups; (C) relative abundance of representative genera in the four groups. The data are shown as the means ± SEM. *, *P* < 0.05; **, *P* < 0.01; ***, *P* < 0.001; ****, *P* < 0.0001.

Principal-coordinate analysis (PCoA) based on Bray-Curtis distance demonstrated that the first principal components explained about 37.57% of the variation, clearly separating WT (WT+NS and WT+ConA) groups and *Gsdmd*^−/−^ (*Gsdmd*^−/−^+NS and *Gsdmd*^−/−^+ConA) groups (Fig. 4A). It indicated that the microbial composition from *Gsdmd*-depleted mice was distinct from that of conventional mice, regardless of ConA administration. We also observed in the PCoA plot that the distance between the WT+NS and WT+ConA groups was greater than that between the *Gsdmd*^−/−^+NS and *Gsdmd*^−/−^+ConA groups, suggesting that *Gsdmd* depletion could partially prevent the microbial dysbiosis caused by ConA injection (Fig. 4A). Analysis of similarity (ANOSIM) and permutational multivariate analysis of variance (PERMANOVA) based on Bray-Curtis distance further corroborated the significantly differential microbial profile between these two different types of mice, and *Gsdmd* knockout to some extent reduced the effect of ConA treatment on the bacterial community (Table S3).

**FIG 5:**
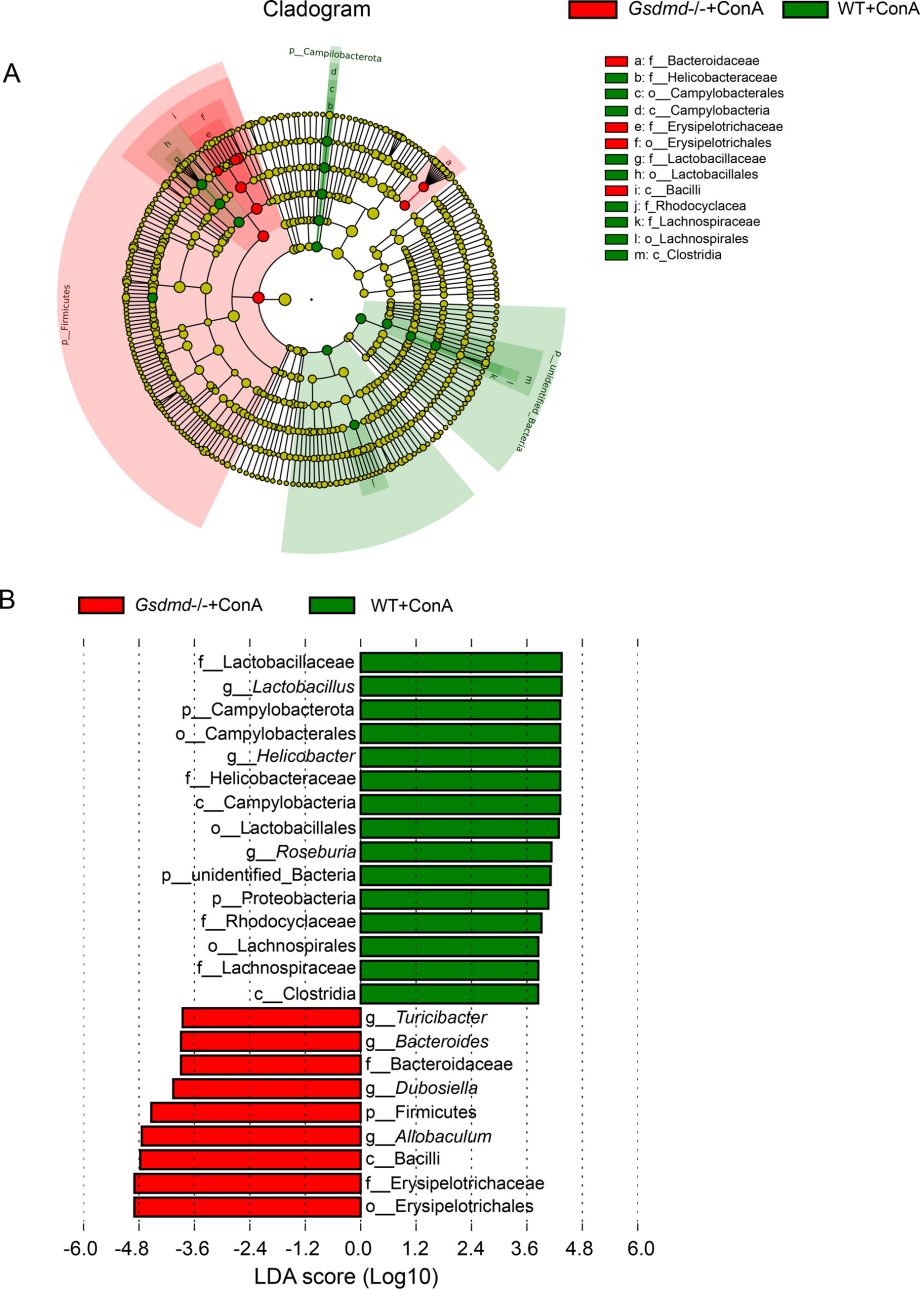
*Gsdmd*-deficient mice reshaped a characteristic gut microbial signature after ConA administration. (A) LEfSe cladogram between the WT+ConA and *Gsdmd*^−/−^+ConA groups; (B) characteristic taxa with linear discriminant analysis (LDA) score of >3.8 in the WT+ConA and *Gsdmd*^−/−^+ConA groups.

The top 10 most abundant taxa at the phylum level were presented in Fig. S1C. The microbial composition demonstrated that WT+NS, WT+ConA, *Gsdmd*^−/−^+NS, and *Gsdmd*^−/−^+ConA groups shared the dominant bacterial phyla, as the *Firmicutes* and *Bacteroidota* phyla accounted for more than 70% of the microbial community in all the four groups. The microbial community at the genus level was dramatically changed in the four groups as illustrated by the compositional diagram of the top 10 most abundant genera (Fig. 4B). The WT+ConA group experienced dramatic decreases in relative abundance of *Allobaculum* and *Dubosiella* compared to the WT+NS group. However, *Gsdmd* knockout in the *Gsdmd*^−/−^+ConA group stabilized the changes in microbes, preventing their reduction (Fig. 4C). Notably, *Allobaculum* and *Lactobacillus* were the two genera most responsive to *Gsdmd* depletion, since the *Gsdmd*^−/−^+NS and *Gsdmd*^−/−^+ConA groups both exhibited a significant higher relative abundance of *Allobaculum* and an obvious lower relative abundance of *Lactobacillus* than their respective control groups (Fig. 4C). Next, linear discriminant analysis (LDA) effect size (LEfSe) analysis at different phylogenetic levels was performed to further explore the characteristic microbes between the *Gsdmd*^−/−^+ConA and WT+ConA groups (Fig. 5A and B). Phylum *Firmicutes*, families *Erysipelotrichaceae* and *Bacteroidaceae*, and genera *Allobaculum*, *Dubosiella*, *Bacteroides*, and *Turicibacter* were enriched in the *Gsdmd*^−/−^+ConA group, whereas the phylum *Campylobacterota*, unidentified_Bacteria, *Proteobacteria*, families *Lactobacillaceae*, *Helicobacteraceae*, *Rhodocyclaceae*, and *Lachnospiraceae*, and genera *Lactobacillus*, *Helicobacter*, and *Roseburia* were enriched in the WT+ConA group.

The 16S rRNA gene sequencing results indicated that removing *Gsdmd* both shaped and stabilized a specific microbiota signature in the *Gsdmd*^−/−^+ConA group, suggesting a contribution toward aggravated liver injury.

### Mice depleted of *Gsdmd* exhibited a distinct fecal metabolic profile after ConA treatment.

**FIG 6:**
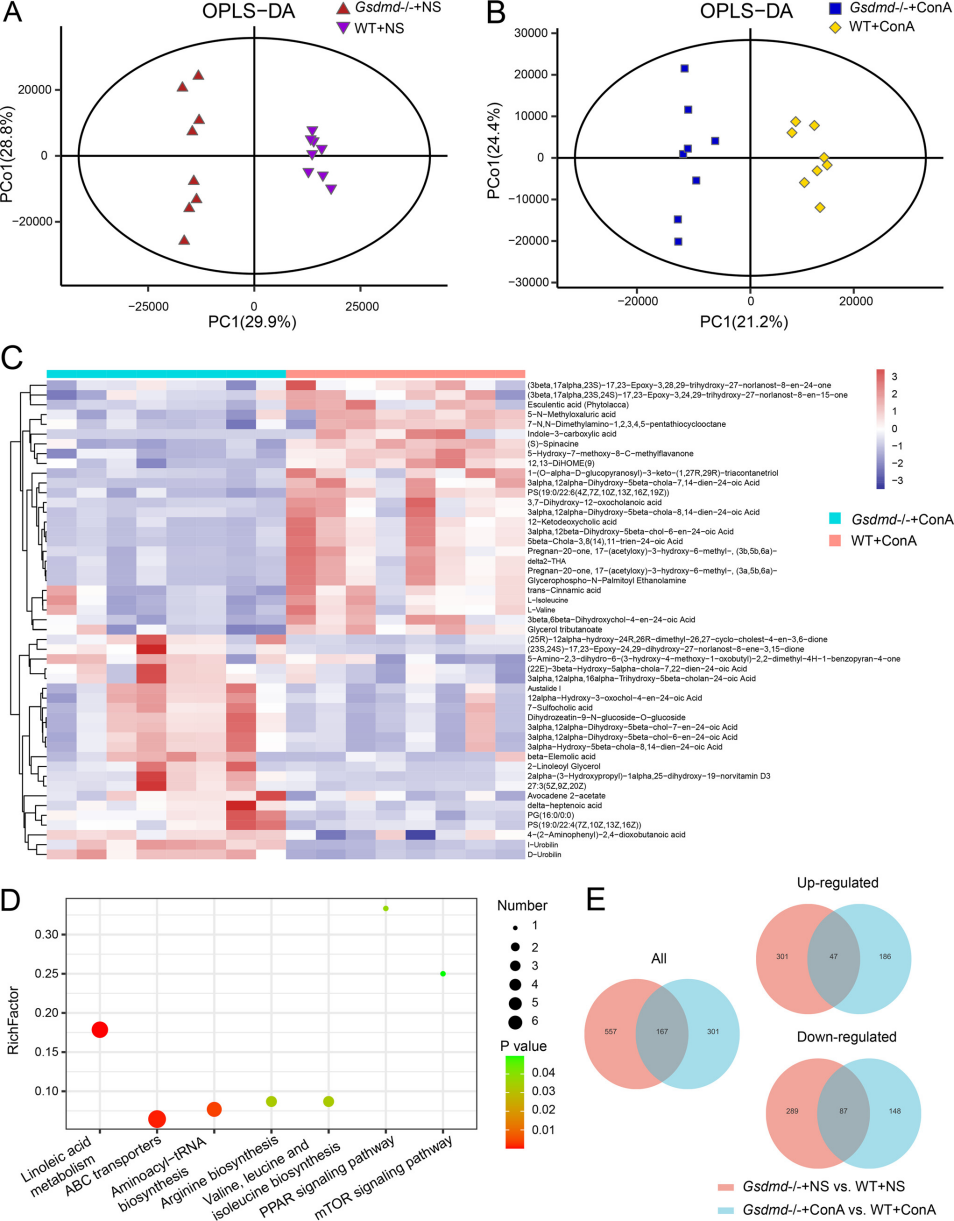
Mice depleted of *Gsdmd* exhibited a distinct fecal metabolic profile after ConA treatment. (A) OPLS-DA plot comparing the fecal metabolite profiles between the WT+NS and *Gsdmd*^−/−^+NS groups; (B) OPLS-DA plot comparing the fecal metabolite profiles between the WT+ConA and *Gsdmd*^−/−^+ConA groups; (C) heatmap showing the top 50 most significantly differentially expressed fecal metabolites between the WT+ConA and *Gsdmd*^−/−^+ConA groups; (D) the significant KEGG pathways enriched by the fecal differential metabolites of the WT+ConA and *Gsdmd*^−/−^+ConA groups; (E) Venn diagram showing the overlap between the fecal differential metabolites of the WT+NS and *Gsdmd*^−/−^+NS groups and those of the WT+ConA and *Gsdmd*^−/−^+ConA groups (all, upregulated, and downregulated).

The fecal metabolome profile was further analyzed using the untargeted liquid chromatography-mass spectrometry (LC-MS) method. As shown in the orthogonal partial least-squares discriminant analysis (OPLS-DA) plot, the WT+NS and *Gsdmd*^−/−^+NS groups were separated into two different clusters [R2X(cum) = 0.643, R2Y(cum) = 0.989, Q2(cum) = 0.909], showing that *Gsdmd* knockout in the basement resulted in a distinct fecal metabolic fingerprint in mice (Fig. 6A). In addition, the WT+ConA and *Gsdmd*^−/−^+ConA groups were also distinguished from each other [R2X(cum) = 0.568, R2Y(cum) = 0.964, Q2(cum) = 0.746] (Fig. 6B). The permutation test (*n* = 200) was performed to validate the reliability of these two prediction models (Q2 = −0.535 and Q2 = −0.58, respectively) (Fig. S2A and B). Based on the criteria of variable importance in the projection (VIP) of >1 and *P* value of <0.05, 724 differential metabolites were found between the WT+NS and *Gsdmd*^−/−^+NS groups, with 348 upregulated and 376 downregulated in the *Gsdmd*^−/−^+NS group compared to the WT+NS group. The top 50 significantly differentiated metabolites were displayed in the heatmap (Fig. S2C). Among the differential metabolites, the most significant class is the triterpenoids with 17/56 upregulated metabolites and 39/56 downregulated metabolites in the *Gsdmd*^−/−^+NS group compared to the WT+NS group. The second richest metabolic class is amino acids, peptides, and analogues with 15/43 elevated metabolites and 28/43 reduced metabolites in the *Gsdmd*^−/−^+NS group compared to the WT+NS group. Following are bile acids, alcohols, and derivatives as the third-largest metabolite class with 13/38 increased metabolites and 25/38 decreased metabolites in the *Gsdmd*^−/−^+NS group compared to the WT+NS group.

There were 468 differential metabolites between the WT+ConA and *Gsdmd*^−/−^+ConA groups. Among these metabolites, 233 were elevated and 235 were decreased in the *Gsdmd*^−/−^+ConA group compared to the WT+ConA group. The hierarchical clustering of the top 50 most significantly differentiated metabolites was shown in Fig. 6C. Among the differential metabolites, the most significant class of these is the fatty acids and conjugates with 17/26 upregulated metabolites and 9/25 downregulated metabolites in *Gsdmd*^−/−^+ConA mice compared to WT+ConA mice. The second richest metabolic class is amino acids, peptides, and analogues with 9/22 elevated metabolites and 13/22 reduced metabolites in *Gsdmd*^−/−^+ConA mice compared to WT+ConA mice. Following are bile acids, alcohols, and derivatives as the third-largest metabolite class with 10/19 increased metabolites and 9/19 decreased metabolites in *Gsdmd*^−/−^+ConA mice compared to WT+ConA mice.

Venn diagram analysis revealed that the differential metabolites in the WT+NS and *Gsdmd*^−/−^+NS groups overlapped those in the WT+ConA and *Gsdmd*^−/−^+ConA groups by 167 metabolites. Among them, 47 were both upregulated and 87 were both downregulated (Fig. 6E). The KEGG pathway enrichment analysis showed that the differential fecal metabolites between the WT+NS and *Gsdmd*^−/−^+NS groups were mainly enriched in metabolic pathways like primary bile acid biosynthesis, sphingolipid metabolism, sphingolipid signaling pathway, etc. (Fig. S2D). Metabolites involved in these pathways are mainly taurine, taurochenodesoxycholic acid, 7-alpha-hydroxycholesterol, etc. (Table S4). The differential metabolites between the WT+ConA and *Gsdmd*^−/−^+ConA groups were significantly enriched in metabolic pathways like linoleic acid metabolism, ABC transporters, aminoacyl-tRNA biosynthesis, arginine biosynthesis, valine, leucine, and isoleucine biosynthesis, peroxisome proliferator-activated receptor (PPAR) signaling pathway, and mTOR signaling pathway (Fig. 6D). Metabolites participating in these pathways are mainly l-glutamine, l-isoleucine, l-valine, *N-*acetyl-d-glucosamine, etc. (Table S4).

Thus, *Gsdmd* depletion reshaped the fecal metabolic profile in mice regardless of ConA treatment. However, the changes in the WT+ConA and *Gsdmd*^−/−^+ConA groups were irrespective of those in the WT+NS and *Gsdmd*^−/−^+NS groups. This indicated some association of the accompanying changes of fecal metabolites in the *Gsdmd*^−/−^+ConA group with the aggravated liver injury.

### *Gsdmd* knockout changed the hepatic metabolic function after the ConA challenge.

**FIG 7:**
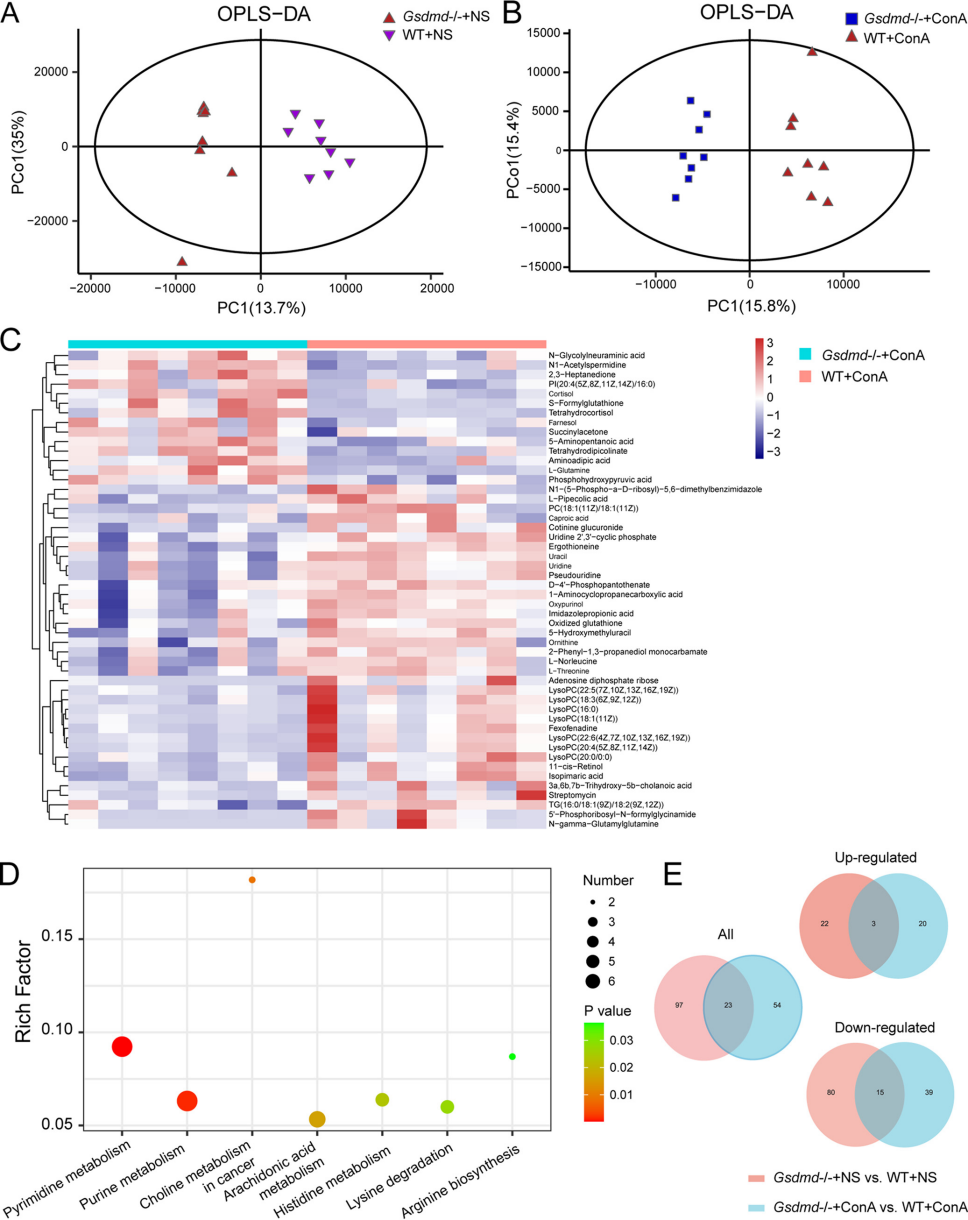
*Gsdmd* knockout changed the hepatic metabolic profile after ConA challenge. (A) OPLS-DA plot comparing the hepatic metabolite profiles between the WT+NS and *Gsdmd^−/−^*+NS groups; (B) OPLS-DA plot comparing the hepatic metabolite profiles between the WT+ConA and *Gsdmd*^−/−^+ConA groups; (C) heatmap showing the top 50 most significantly differentially expressed hepatic metabolites between the *Gsdmd*^−/−^+ConA and WT+ConA groups; (D) the significant KEGG pathways enriched by the hepatic differential metabolites of the *Gsdmd*^−/−^+ConA and WT+ConA groups; (E) Venn diagram showing the overlap between the liver differential metabolites of the WT+NS and *Gsdmd*^−/−^+NS groups and those of the WT+ConA and *Gsdmd*^−/−^+ConA groups (all, upregulated, and downregulated).

Next, we tested the hepatic metabolic profile between the four groups. The OPLS-DA plot depicted a clear separation of hepatic metabolic profile between the WT+NS and *Gsdmd*^−/−^+NS groups; the same was true for the WT+ConA and *Gsdmd*^−/−^+ConA groups [R2X (cum) = 0.61, R2Y(cum) = 0.928, Q2(cum) = 0.554; R2X(cum) = 0.416, R2Y(cum) = 0.957, Q2(cum) = 0.331, respectively) (Fig. 7A and B). The permutation test (*n* = 200) showed good reliability of these two prediction models (Q2 = −0.499; Q2 = −0.558, respectively) (Fig. S3A and B). Based on the criteria of VIP of >1 and *P* value of <0.05, 120 metabolites were significantly different between the WT+NS and *Gsdmd*^−/−^+NS groups, with 25 metabolites elevated and 95 metabolites decreased in the *Gsdmd*^−/−^+NS group compared to the WT+NS group. The top 50 significantly differentiated metabolites between the WT+NS and *Gsdmd*^−/−^+NS groups were displayed in the heatmap (Fig. S3C). These metabolites mainly belonged to amino acids, peptides, and analogues (35/37 downregulated and 2/37 upregulated in the *Gsdmd*^−/−^+NS group) and carbohydrates and carbohydrate conjugates (12/14 downregulated and 2/14 upregulated in the *Gsdmd*^−/−^+NS group). In the *Gsdmd*^−/−^+ConA group, 23 metabolites were upregulated and 54 metabolites were downregulated compared to the WT+ConA group. The heatmap showed the top 50 significantly differentiated metabolites between the WT+ConA and *Gsdmd*^−/−^+ConA groups (Fig. 7C). Among the differential metabolites, the most prominent changed metabolites belonged to amino acids, peptides, and analogues (7/19 upregulated and 12/19 downregulated in the *Gsdmd*^−/−^+ConA group). Following is the glycerophosphocholine class; interestingly, all 11 metabolites belonging to this class, that is, PC(18:1(11Z)/18:1(11Z)), LysoPC(16:0), LysoPC(22:6(4Z,7Z,10Z,13Z,16Z,19Z)), LysoPC(20:4(5Z,8Z,11Z,14Z)), LysoPC(18:1(11Z)), LysoPC(18:3(6Z,9Z,12Z)), LysoPC(20:0/0:0), LysoPC(22:5(7Z,10Z,13Z,16Z,19Z)), LysoPC(20:1(11Z)), LysoPC(20:4(8Z,11Z,14Z,17Z)), and LysoPC(15:0), were downregulated in the *Gsdmd*^−/−^+ConA group in comparison with the WT+ConA group.

Venn diagram analysis revealed that the differential metabolites between the WT+NS and *Gsdmd*^−/−^+NS groups have 23 overlaps with those between the WT+ConA and *Gsdmd*^−/−^+ConA groups, among which 3 were both upregulated and 15 were both downregulated (Fig. 7E). Notably, *Gsdmd* depletion reduced the level of l-glutamine in the *Gsdmd*^−/−^+NS group compared to the WT+NS group, whereas ConA treatment increased its level in the *Gsdmd*^−/−^+ConA group compared to the WT+ConA group, indicating an involvement of l-glutamine in the response to ConA in *Gsdmd*^−/−^ mice.

The KEGG pathway enrichment analysis showed that the differential metabolites between the WT+NS and *Gsdmd*^−/−^+NS groups were mainly enriched in metabolic pathways like aminoacyl-tRNA biosynthesis, regulation of lipolysis in adipocytes, purine metabolism, and cysteine and methionine metabolism (Fig. S3D). Metabolites involved in these pathways are mainly l-glutamine, adenosine monophosphate (AMP), l-serine, etc. (Table S4). The differential metabolites between the WT+ConA and *Gsdmd*^−/−^+ConA groups were significantly enriched in metabolic pathways like pyrimidine metabolism, purine metabolism, choline metabolism in cancer, arachidonic acid metabolism, histidine metabolism, lysine degradation, and arginine biosynthesis (Fig. 7D). Metabolites participating in these pathways are mainly PC(18:1(11Z)/18:1(11Z)), 5-aminopentanoic acid, l-glutamine, imidazolepropionic acid, uridine, etc. (Table S4).

The results showed that the differential metabolites and enriched pathways in the WT+ConA and *Gsdmd*^−/−^+ConA groups were independent of those in the WT+NS and *Gsdmd*^−/−^+NS groups, suggesting the contribution of changed liver metabolic profile in the *Gsdmd*^−/−^+ConA group to the aggravation of liver injury.

Notably, when comparing differential liver and fecal metabolites between the WT+ConA and *Gsdmd*^−/−^+ConA groups, we found that the levels of l-glutamine and N1-acetylspermidine were increased in the liver but decreased in the feces of the *Gsdmd*^−/−^+ConA group compared to WT+ConA group. The level of l-pipecolic acid was decreased while succinylacetone was increased in both liver and feces in the *Gsdmd*^−/−^+ConA group compared to WT+ConA group. Interestingly, l-glutamine and its involved arginine biosynthesis are the common metabolite enrichment pathway in liver and feces.

### Correlations between liver injury indexes, key inflammatory indicators, intestinal barrier indexes, representative microbes, and characteristic metabolites.

**FIG 8:**
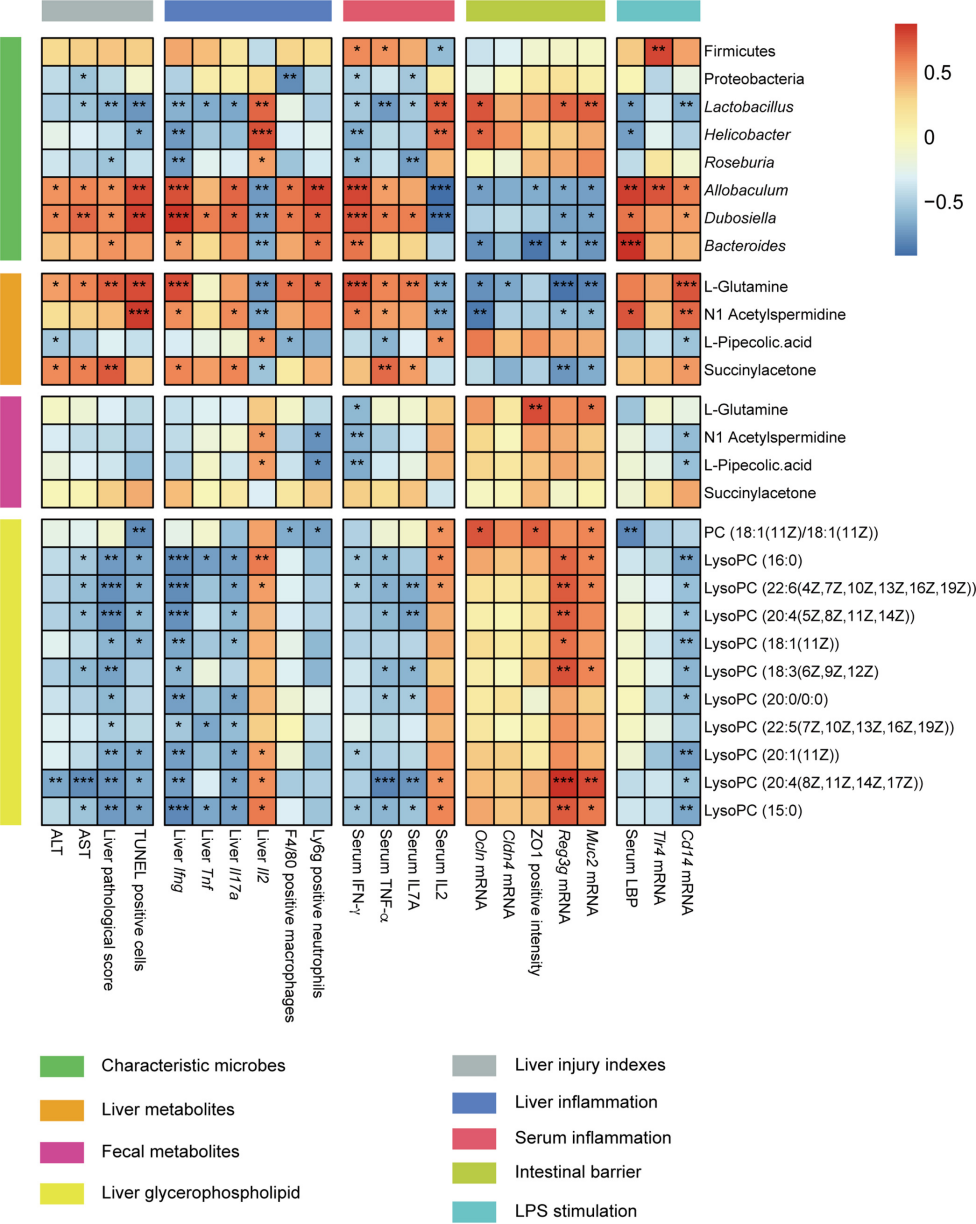
Correlations heatmap of the liver injury indexes, inflammatory cytokines, intestinal barrier index, representative microbes, characteristic metabolites, and other representative biomarkers. The color scale indicates the magnitude of the correlation: red indicates a positive correlation and blue indicates a negative correlation. Asterisks indicate significance: *, *P* < 0.05; **, *P* < 0.01; ***, *P* < 0.001.

Spearman correlation analysis was used to assess the correlation between liver injury indicators, hepatic and serum inflammatory factors, intestinal barrier damage parameters, hepatic LPS stimulation indexes, characteristic bacteria, and hepatic metabolites (Fig. 8). Certain microbes were closely associated with liver injury, inflammation, and intestinal barrier function. *Lactobacillus* was negatively correlated with AST, liver pathological score, percentage of TUNEL-positive cells, hepatic expressions of *Ifng*, *Tnf*, and *Il17a*, serum levels of IFN-γ, TNF-α, and IL17A, levels of serum LBP, and expression of hepatic *Cd14*. The relative abundances of *Lactobacillus* spp. had positive correlations with expressions of *Ocln*, *Muc2*, and *Reg3g*. The relative abundance of *Roseburia* spp. was negatively related to liver pathological score, hepatic expression of *Ifng*, and serum levels of IFN-γ and IL17A. *Allobaculum* spp. and *Dubosiella* spp. are two bacteria whose abundances significantly increased after *Gsdmd* depletion. Their relative abundances were positively associated with levels of liver injury indexes (ALT and AST), hepatic pathological score, percentage of hepatic apoptosis cells, hepatic expressions of *Ifng*, *Tnf*, and *Il17a*, serum levels of key inflammatory cytokines (IFN-γ, TNF-α, and IL17A), percentages of F4/80- and Ly6G-positive inflammatory cells, serum levels of LBP, and hepatic expressions of *Tlr4* and *Cd14*. However, *Allobaculum* spp. were negatively correlated with the transcriptional levels of *Ocln*, *Reg3g*, and *Muc2* and fluorescence intensity of ZO-1. *Dubosiella* spp. were negatively correlated with colonic expression levels of *Reg3g* and *Muc2.* The relative abundance of *Bacteroides* spp. was negatively correlated with intestinal expressions of *Ocln*, *Reg3g*, and *Muc2* and positive strength of ZO-1.

Changes in the hepatic metabolites are also closely related to liver injury, inflammation, and intestinal barrier function. Liver l-glutamine was positively correlated with levels of ALT and AST, liver pathological score, percentage of TUNEL-positive cells, hepatic expression of *Ifng*, serum levels of IFN-γ, TNF-α, and IL-17A, increased infiltrating F4/80^+^ macrophages and Ly6G^+^ neutrophils, and hepatic expression of *Cd14* but negatively correlated with expressions of *Ocln*, *Cldn4*, *Reg3g*, and *Muc2*. Glycerophospholipid metabolites (phosphatidylcholine [PC] and lysoPCs) are negatively correlated with levels of AST, liver pathological score, percentage of hepatic apoptosis cells, hepatic expressions of *Ifng*, *Tnf*, and *Il17a*, serum levels of IFN-γ, TNF-α, and IL17A, and levels of *Cd14* and positively correlated with expressions of *Reg3g* and *Muc2*. However, the characteristic fecal metabolites did not show significant correlations with liver injury parameters, inflammation, and intestinal barrier indexes.

## DISCUSSION

AIH is a progressive inflammation-associated liver injury. Pyroptosis is a novel inflammatory programmed cell death, and its role in AIH has not been well studied yet. GSDMD, which serves as the executioner of pyroptosis, has been reported to be involved in several liver diseases. Nevertheless, the actual role of GSDMD in AIH remains to be revealed. In this study, after 8 h of ConA challenge, *Gsdmd*^−/−^ mice exhibited more severe liver damage than WT mice, which was characterized by a lower survival rate, more extensive hepatocyte necrosis and apoptosis, and higher serum transaminase levels. These results established a protective effect of GSDMD on ConA-induced autoimmune liver injury.

The gut-liver axis has been revealed to be involved in AIH because the translocation of LPS from a leaky gut could activate inflammation and promote the progression of liver injury (21, 24). We interestingly found that compared to WT mice, *Gsdmd*^−/−^ mice showed intestinal barrier impairment in the absence of ConA treatment as indicated by decreased colonic expressions of *Tjp1*, *Ocln*, *Cldn4*, *Reg3g*, and *Muc2* and a slightly increased serum level of LBP. It indicated a protective role of GSDMD on the intestinal barrier under physiological conditions. Consistently, an unexpected physiological nonpyroptosis function of GSDMD in maintaining the integrity of the intestinal mucosal barrier has been described by Zhang et al. (28). They observed depletion of the mucus layer in mice with either whole-body or intestinal epithelial cell (IEC)-specific *Gsdmd* knockout, and these mice were more susceptible to enteric pathogen infection (28). But the disruption of the intestinal mucosal barrier caused by *Gsdmd* knockout was not sufficient to cause liver injury in the absence of ConA treatment. This was due to the self-protective ability of the liver under steady-state conditions. However, after ConA treatment, *Gsdmd*^−/−^ mice exhibited more pronounced liver damage and inflammatory infiltration, accompanied by endotoxin influx after barrier damage. With the upregulation of *Tlr4* and *Cd14* in the *Gsdmd*^−/−^+ConA group, an increase in LPS stimulation in the liver could be observed. Furthermore, the *Gsdmd*^−/−^+ConA group showed higher systemic levels of IFN-γ, TNF-α, and IL17A and liver-specific expressions of *Ifng*, *Tnf*, and *Il17a* than the WT+ConA group. IFN-γ, TNF-α, and IL-17 were key inflammatory cytokines critically involved in the pathogenesis of ConA-induced liver injury (29–31). Functional deficiency of IFN-γ or antagonization of TNF-α in mice significantly inhibited the progression of the disease (29, 30, 32). In contrast, many studies on GSDMD have obtained results of reduced production of inflammatory cytokines and relieved severe liver inflammation, which seem to contradict our findings (8, 33). However, other research by Shi et al. also demonstrated the role of GSDMD in protecting the intestinal barrier and restraining inflammation (34). They found that *Gsdmd*^−/−^ mice fed on a high-fat diet (HFD) showed higher serum levels of inflammation cytokines (TNF-α, monocyte chemoattractant protein 1 [MCP-1], and IL-6), lower levels of TJ proteins (ZO-1, occludin, and claudin), and significantly higher endotoxemia than the corresponding control WT mice. Our work suggested the bidirectional interaction between intestinal barrier damage and ConA-induced liver injury. It may be the increased production of key inflammatory cytokines and hepatic inflammation stimulation caused by translocated LPS from the leaky gut aggravated the liver damage in ConA-treated *Gsdmd*^−/−^ mice.

Intestinal commensal microbes essentially contribute to the intestinal barrier and its immune system (35, 36). Intriguingly, the expression and activation of GSDMD depend on the commensal gut microbiota (28). Our results showed that *Gsdmd* knockout shaped an intestinal microbial structure distinct from that of conventional mice, regardless of ConA administration. ConA-induced AIH triggered microbial dysbiosis in the WT+ConA group compared to the WT+NS group. However, *Gsdmd* ablation reduced the intestinal microbial dysbiosis since the *Gsdmd*^−/−^+ConA group shared a similar microbial composition with the *Gsdmd*^−/−^+NS group. Thus, the specific microbiota signature in the *Gsdmd*^−/−^+ConA group might contribute to the aggravated liver injury. The absence of *Gsdmd* decreased the relative abundance of *Lactobacillus* spp. and *Roseburia* spp. while increasing the relative abundance of *Allobaculum* spp. The *Lactobacillus* genus could protect the integrity of the intestinal mucosal barrier by promoting intestinal epithelial proliferation (37, 38). Consistently, our work found a positive correlation between *Lactobacillus* spp. and intestinal TJ parameters, mucin, and antimicrobial peptide. *Roseburia* spp. were also reported to be able to improve gut homeostasis and maintain the intestinal barrier (39, 40). Unfortunately, we did not observe an association between *Roseburia* spp. and gut barrier parameters in this study. In addition, a negative relationship between *Allobaculum* spp. and intestinal barrier (*Tjp1*, *Ocln*, *Muc2*, and *Reg3g*) has been demonstrated. One species from the *Allobaculum* genus, *Allobaculum mucolyticum*, was reported as an inflammatory bowel disease (IBD)-associated species since its mucin-degrading property contributes to the pathogenesis of IBD (41). Other researchers have reported that bacterial signals which induced mucus secretion and maintained the intestinal barrier were dependent on GSDMD (28). Hence, the significant increase of the mucolytic bacterium caused by *Gsdmd* depletion in our study indicated a possible link between GSDMD and mucin degradation, besides mucin secretion.

*Lactobacillus* spp. were negatively correlated with liver injury indexes, key inflammatory cytokines, and endotoxemia, indicating a beneficial role of commensal *Lactobacillus* spp. in host hepatic health. Lactobacillus reuteri is a well-recognized probiotic belonging to the *Lactobacillus* genus. It has been reported that supplementation with L. reuteri significantly ameliorated inflammation and improved the outcomes of other liver disorders (42–44). However, it is still unknown whether L. reuteri had protective effects on ConA-induced AIH. The relative abundance of *Allobaculum* spp. had strongly positive correlations with liver damage parameters, hepatic LPS stimulation, and inflammation response. Miyauchi et al. reported the ability of an *Allobaculum* strain (OTU002) to induce the expansion of inflammatory intestinal T helper 17 cells and contaminant cytokine production of IL-17 in experimental autoimmune encephalitis (45). Although few studies have elucidated the exact role of *Allobaculum* spp., the interactions between *Allobaculum* spp. and immune cells and the bacterium’s involvement in autoimmune diseases are worth investigating. Interestingly, flagellin of Roseburia intestinalis, one of the most widely studied species in the *Roseburia* genus, has been demonstrated to decrease the cleavage of GSDMD and inhibit inflammasome-triggered pyroptosis (46). In this light, the relationship between *Roseburia* spp. and GSDMD or pyroptosis deserves further investigation. So, here we have a hypothesis that changes in the abundance of specific microbes caused by *Gsdmd* deletion might affect the intestinal barrier and further contribute to the progression of ConA-induced liver injury. Research on whether GSDMD affects disease through the intestinal microbiome is still in its infancy, but it is full of promise. Some researchers demonstrated a noncritical role of the gut microbial community as cohousing with WT mice did not change the impact of *Gsdmd* depletion on colitis (47, 48), while others suggested that the gut microbiome partially mediated the effect of GSDMD in HFD-fed mice (34). Collectively, our results provide a preliminary point of the involvement of the gut microbes in the aggravation of liver injury in the *Gsdmd*^−/−^+ConA group.

The results of liver metabolomic analysis indicated that although the metabolic profiles between the WT+NS and *Gsdmd*^−/−^+NS groups were different, as were those of the WT+ConA and *Gsdmd*^−/−^+ConA groups, the differential metabolites between NS groups and ConA groups were mostly independent of each other. Therefore, it may be the accompanying changes in hepatic metabolites in the *Gsdmd*^−/−^+ConA group that are associated with the progression of liver injury. Large quantities of hepatic metabolites dysregulated in the *Gsdmd*^−/−^+ConA group were involved in energy metabolism, like fatty acids and amino acids. Glutamine is considered a “fuel for the immune system,” and it is an essential nutrient for a large number of immune cells, such as lymphocytes, macrophages, and neutrophils (49–51). Previous studies have showed the involvement of glutamine in ConA-induced lymphocyte activation and cytokine production (52). And here, we found an increase of hepatic l-glutamine in the *Gsdmd*^−/−^+ConA group and further described a positive correlation of l-glutamine with liver injury indexes, hepatic expressions of *Ifng*, and recruitment of macrophages and neutrophils as well as systemic inflammation. This suggested that the accumulation of glutamine in the liver may be a response to the increased nutritional demands resulting from the aggravated inflammatory response in the *Gsdmd*^−/−^+ConA group. Importantly, downregulation of glycerophospholipid metabolism was found in ConA-treated *Gsdmd*^−/−^ mice as reflected by the significant decreases of 11 glycerophospholipid metabolites (primary PC and lysoPCs). The liver is the main organ for lysoPC biogenesis (53), and lysoPCs regulate a variety of biological processes, including cell proliferation and inflammation (54). These downregulated glycerophospholipid metabolites negatively correlated with liver injury and inflammation indexes. Consistently, others demonstrated that glycerophospholipids (lysoPCs and lysophosphatidylethanolamines [lysoPEs]) were highly correlated with the outcomes of ConA-induced liver injury and could be a potential biomarker for autoimmune liver injury (55). Thus, we proposed the importance of glycerophospholipid metabolism to the progression of ConA-induced hepatitis in *Gsdmd*^−/−^ mice.

Nevertheless, the mice we used were a whole-body *Gsdmd* knockout, which left some questions. Some researchers observed that hepatocytes are insensitive to pyroptosis, since GSDMD blockade in the innate immune cell protects against hepatic ischemia-reperfusion injury but the same was not true for hepatocytes (56, 57). In contradiction, others demonstrated an essential role of GSDMD-mediated hepatocyte pyroptosis in acute liver failure (58). More research is needed to elaborate on the decisive cell populations for the protectiveness of GSDMD in AIH. In addition, since we proposed a crucial role of the intestinal barrier in the aggravation of AIH in *Gsdmd*^−/−^ mice, intestinal epithelial cell (IEC)-knockout mice could also be used to explore the causal relationship between GSDMD, the intestinal barrier, and AIH. Furthermore, the direct contribution of the altered hepatic metabolites to AIH also needs further investigation.

### Conclusion.

Our work demonstrated that the complete absence of *Gsdmd* in mice exacerbated ConA-induced autoimmune liver injury, providing the first direct clues to the protectiveness of GSDMD in AIH. A damaged intestinal barrier and changes in certain microbes may play the major role in the aggravation of liver injury in *Gsdmd*^−/−^ mice. Our work affirms the significant physiological effect of GSDMD on the intestinal barrier as well as its interaction with the gut microbiota besides being the executioner of pyroptosis. The role of GSDMD in autoimmune liver diseases or other liver diseases is complex and intriguing, deserving deep investigation.

## MATERIALS AND METHODS

### Animals and ConA-induced hepatoxicity.

*Gsdmd*^−/−^ mice were purchased from the Model Animal Research Center of Nanjing University and bred in our facility. Mice aged 6 to 8 weeks were used in this experiment. Littermate WT mice were used as a control for genetic knockout mice. Mice were divided into four groups: WT+NS, WT+ConA, *Gsdmd*^−/−^+NS, and *Gsdmd*^−/−^+ConA. Mice in the ConA group were challenged with 15 mg/kg ConA (Sigma-Aldrich) through tail vein injection to establish AIH. Mice in the NS group received an equal amount of 0.9% normal saline (NS). Mice were sacrificed 8 h after treatment, and feces, blood, liver, and colon tissues were collected. All experiments were approved by the Animal Care and Use Committee of the First Affiliated Hospital, School of Medicine, Zhejiang University.

### ALT and AST assessment.

Serum alanine aminotransferase (ALT) and aspartate transaminase (AST) levels were measured using the Beckman Coulter AU5800 chemistry system.

### Histology analysis.

Liver and colon tissues were fixed in 4% paraformaldehyde fixative solution immediately after collection and then embedded in paraffin. The paraffin-embedded samples were cut into 3-μm sections. Liver sections were stained with hematoxylin and eosin (H&E) and observed under a light microscope. The degree of hepatic pathological damage was scored according to the inflammation of the portal area and lobule and fusion necrosis. Hepatocyte apoptosis was detected by the TUNEL BrightGreen apoptosis detection kit (Vazyme, A112) according to the instructions. For immunohistochemistry, liver sections were stained with F4/80 (macrophage) and Ly6G (neutrophils). Immunofluorescence staining was used to observe the expression of intestinal barrier marker ZO-1 in the colonic epithelium of mice in each group. Positive cells were counted using Image J software.

### Serum cytokine assessment.

The concentrations of serum cytokines were determined by a 23-plex assay kit (Bio-Plex Pro mouse cytokine 23-Plex panel; Bio-Rad, Hercules, CA, USA) and analyzed using the Magpix system (Luminex Corporation) and Bio-Plex Manager 6.1 software (Bio-Rad) according to the manufacturer’s instructions.

### RNA extraction and real-time PCR analysis.

Total RNA was extracted from the liver and colon tissues using the RNeasy Plus minikit (Qiagen, Hilden, Germany) according to the manufacturer’s instructions. Extracted RNA was immediately reverse transcribed to cDNA and stored at −80°C until use. mRNA relative abundances of targeted genes were measured in duplicate with *Gapdh* serving as the internal reference, and threshold cycle (ΔΔ*C_T_*) was finally calculated to analyze the significant difference between groups (see Table S5 in the supplemental material for primer information).

### Serum LBP analysis.

A mouse LBP enzyme-linked immunosorbent assay (ELISA) kit (LPS binding protein) (Abcam, ab269542) was used for quantification of serum LBP concentration according to the manufacturer’s protocol.

### 16S rRNA gene sequencing.

Total bacterial genomic DNA was extracted from feces using the DNeasy PowerSoil Pro kit (Qiagen, Hilden, Germany) according to the manufacturer’s protocol. The V3-V4 region of the 16S rRNA gene was amplified using universal bacterial primers with sample-specific barcodes (341F, 5′-CCTAYGGGRBGCASCAG-3′, and 806R, 5′-GGACTACNNGGGTATCTAAT-3′). The amplified products were detected by electrophoresis on a 2% agarose gel and then recycled and purified with the Qiagen gel extraction kit (Qiagen, Hilden, Germany). Sequencing libraries were generated using the TruSeq DNA PCR-free sample preparation kit (Illumina, USA) following the manufacturer’s recommendations. The library quality was assessed on the Qubit 2.0 fluorometer (Thermo Scientific, USA) and the Agilent Bioanalyzer 2100 system. At last, the qualified library was sequenced on a NovaSeq 6000 (PE 250) platform. Raw sequencing reads with exact matches to the barcodes were assigned to the respective samples and identified as valid sequences. Paired-end reads were merged using FLASH (V1.2.7), and then filtered under specific filtering conditions to obtain the high-quality clean sequences according to the QIIME (V1.7.0) quality-controlled process. The rarefaction curve was drawn to evaluate the sequencing depth and rationality (from 10 to 27,922, total of 6 steps with 4,652 sequences per step). Then, after the chimera removal, the effective tags were clustered into operational taxonomic units (OTUs) at 97% sequence identity using Usearch software (Usearch v10). For each representative sequence, the GreenGene database was used based on the RDP classifier (version 2.2) to annotate the taxonomic information of each representative sequence within each OTU. Then, the statistical analysis of microbial diversity and differential enrichment was conducted.

### Fecal LC-MS metabolome assessment.

Sixty milligrams of6 stool was mixed thoroughly with 20 μL l-2-chlorophenylalanine which served as the internal standard and 600 μL methanol-water (4:1, vol/vol) and allowed to stand at −20°C for 5 min. After grinding at 60 Hz for 2 min, the mixture was treated ultrasonically for 10 min and then allowed to stand at −20°C for another 30 min. After that, the mixture was centrifuged for 10 min at 13,000 rpm at 4°C and 200 μL supernatant was transferred to the sample vial and dried, then resuspended in 300 μL methanol-water (4:1, vol/vol), vortexed, ultrasonicated for 3 min, and allowed to stand at −20°C for another 2 h. After that, the mixture was centrifuged for 10 min at 13,000 rpm, 4°C, and the supernatant was filtered through a 0.22-μm filter and transferred into the sample vial for LC-MS analysis in a Dionex Ultimate 3000 RS ultrahigh-performance LC (UHPLC) system fitted with a Q-Exactive quadrupole-Orbitrap mass spectrometer.

The acquired LC-MS raw data were analyzed by Progenesis QI software (Waters Corporation, Milford, MA, USA). Metabolites were identified by Progenesis QI (Waters Corporation, Milford, MA, USA) data processing software, based on public databases and self-built databases. Orthogonal partial least-squares discriminant analysis (OPLS-DA) was carried out to visualize the metabolic profiling among experimental groups. Hotelling’s T2 region, shown as an ellipse in score plots of the models, defines the 95% confidence interval of the modeled variation. Variable importance in the projection (VIP) ranks the overall contribution of each variable to the OPLS-DA model, and those variables with VIP values of >1 are considered relevant for group discrimination. The differential metabolites were selected based on a VIP value of >1 and *P* values of <0.05.

### Liver LC-MS metabolome assessment.

Thirty milligrams of liver tissues was mixed thoroughly with 20 μL l-2-chlorophenylalanine which served as the internal standard and 400 μL methanol-water (4:1, vol/vol) and precooled at −20°C for 2 min. After grinding at 60 Hz for 2 min, the mixture was processed ultrasonically in an ice bath for 10 min and then allowed to stand at −20°C for another 30 min. After that, the mixture was centrifuged for 10 min at 13,000 rpm at 4°C and 300 μL supernatant was transferred to the sample vial. After being dried, it was resuspended in 300 μL methanol-water (4:1, vol/vol), ultrasonicated for 3 min, and allowed to stand at −20°C for another 2 h. After that, the mixture was centrifuged for 10 min at 13,000 rpm, 4°C, and 150 μL supernatant was filtered through a 0.22-μm filter and transferred into the sample vial for LC-MS analysis in a Dionex Ultimate 3000 RS UHPLC system fitted with a Q-Exactive quadrupole-Orbitrap mass spectrometer.

The acquired LC-MS raw data were analyzed by the Progenesis QI software (Waters Corporation, Milford, MA, USA). Metabolites were identified by Progenesis QI (Waters Corporation, Milford, MA, USA) data processing software, based on public databases and self-built databases. Orthogonal partial least-squares discriminant analysis (OPLS-DA) was carried out to visualize the metabolic profiling among experimental groups. Hotelling’s T2 region, shown as an ellipse in score plots of the models, defines the 95% confidence interval of the modeled variation. VIP ranks the overall contribution of each variable to the OPLS-DA model, and those variables with a VIP value of >1 are considered relevant for group discrimination. The differential metabolites were selected based on a VIP value of >1 and a *P* value of <0.05.

### Statistical analysis.

Statistical analyses were performed using the statistical computer package GraphPad Prism (version 8) and R language (R 3.6.3). Results are expressed as means ± standard errors of the means (SEM). Statistical comparisons were made using one-way ANOVA with Tukey’s *post hoc* test or Student’s *t* test or the Mann-Whitney U test when appropriate. Differences were considered significant at a *P* value of <0.05.

### Data availability.

All data generated or analyzed during this study are included in this published article and its supplemental material. The data sets generated during the current study are available in the GenBank Sequence Read Archive repository under BioProject identifier (ID) PRJNA855142.
