# Supplementary material for: Multi-Omics Analysis Reveals the Protection of Gasdermin D in Concanavalin A-Induced Autoimmune Hepatitis
Source: Microbiol Spectr. 2022 Aug 16;10(5):e01717-22. doi: 10.1128/spectrum.01717-22 (PMC9602755; doi:10.1128/spectrum.01717-22)
Supplement: Supplemental file 1 — Supplemental material. Download spectrum.01717-22-s0001.pdf, PDF file, 0.7 MB [file spectrum.01717-22-s0001.pdf]

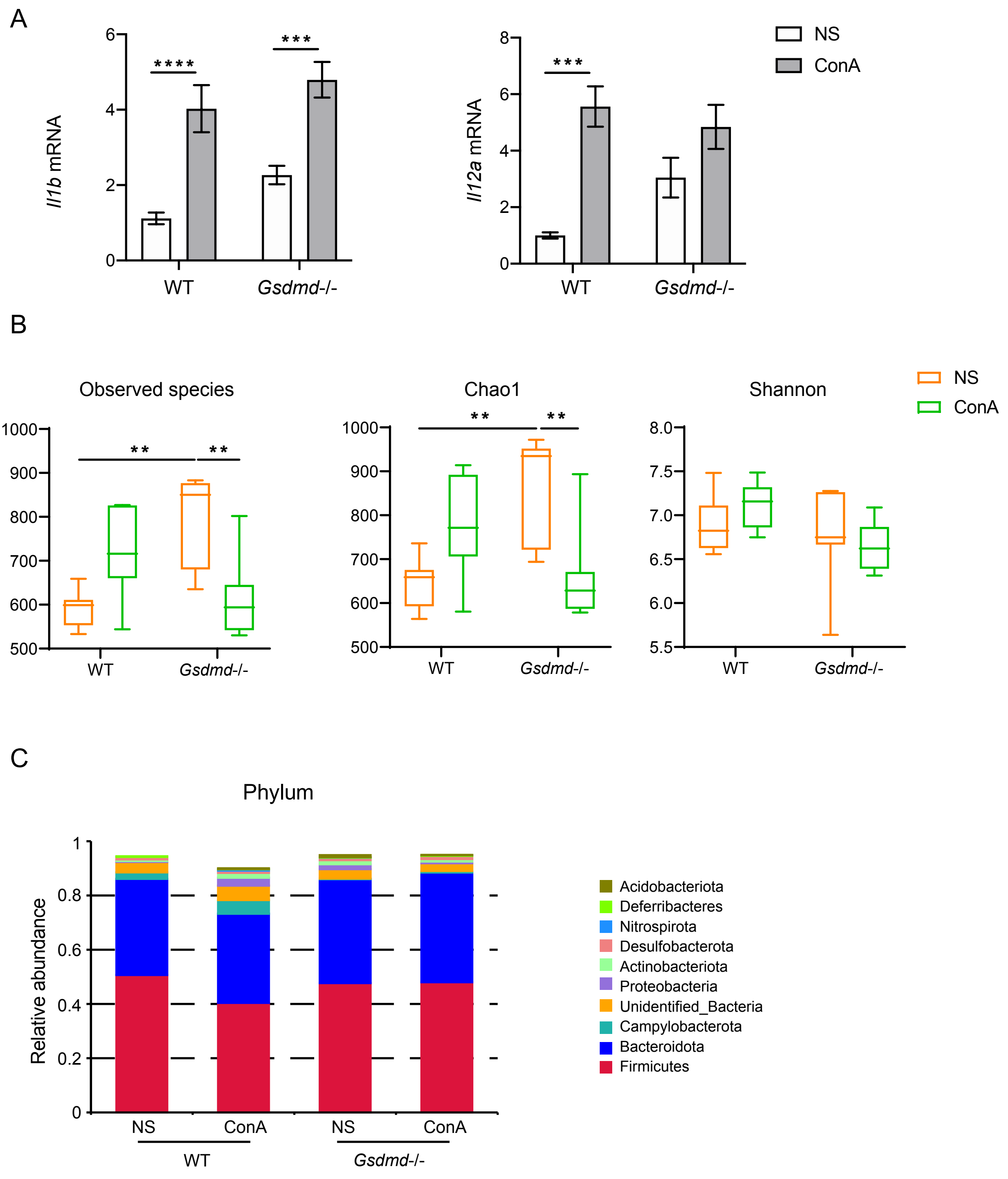
A
Il1b mRNA
Il12a mRNA
NS
ConA
WT
Gsdmd-/-
****
***
B
Observed species
Chao1
Shannon
NS
ConA
**
WT
Gsdmd-/-
C
Phylum
Relative abundance
Acidobacteriota
Deferribacteres
Nitrospirota
Desulfobacterota
Actinobacteriota
Proteobacteria
Unidentified_Bacteria
Campylobacterota
Bacteroidota
Firmicutes
NS
ConA
NS
ConA
WT
Gsdmd-/-

Figure S1

Figure S1

A. Hepatic transcriptional levels of *Il1b* and *Il12a* in the four groups, data are shown as the mean ± SEM. *, $P<0.05$; **, $P<0.01$; ***, $P<0.001$; ****, $P<0.0001$;

B. α-diversity indexes (observed species, chao1, and Shannon) of the fecal microbiota in the four groups, data are given as median with the interquartile range and both the maximum and minimum values, *, $P<0.05$; **, $P<0.01$; ***, $P<0.001$; ****, $P<0.0001$;

C. Histogram of the top 10 most abundant phylum of the fecal microbiota in the four groups.

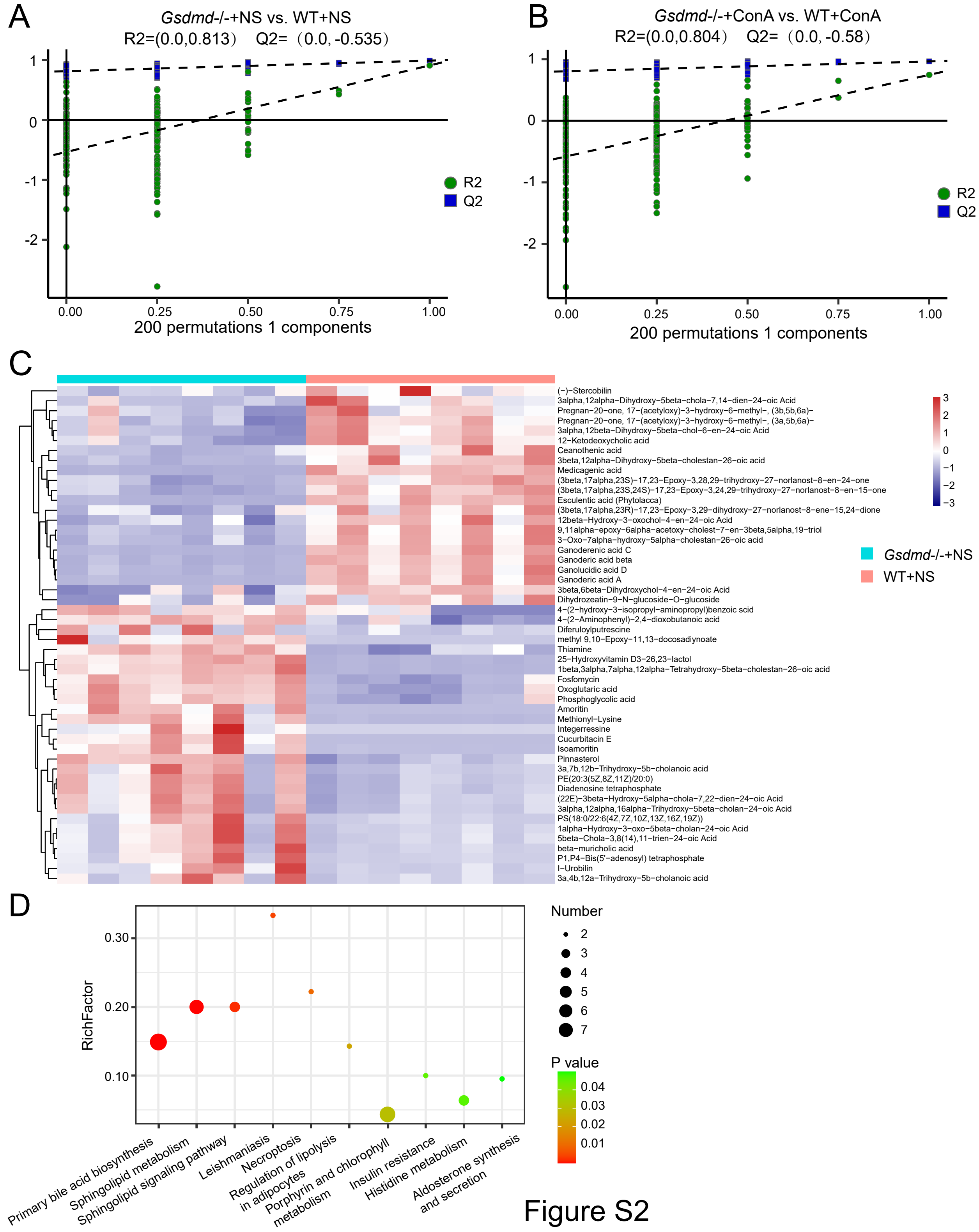

A
Gsdmd-/-+NS vs. WT+NS
R2=(0.0,0.813) Q2= (0.0, -0.535)
R2
Q2
200 permutations 1 components
B
Gsdmd-/-+ConA vs. WT+ConA
R2=(0.0,0.804) Q2= (0.0, -0.58)
R2
Q2
200 permutations 1 components
C
(-)-Stercobilin
3alpha,12alpha-Dihydroxy-5beta-chola-7,14-dien-24-oic Acid
Pregnan-20-one, 17-(acetyloxy)-3-hydroxy-6-methyl-, (3b,5b,6a)-
Pregnan-20-one, 17-(acetyloxy)-3-hydroxy-6-methyl-, (3a,5b,6a)-
3alpha,12beta-Dihydroxy-5beta-chol-6-en-24-oic Acid
12-Ketodeoxycholic acid
Ceanothenic acid
3beta,12alpha-Dihydroxy-5beta-cholestan-26-oic acid
Medicagenic acid
(3beta,17alpha,23S)-17,23-Epoxy-3,28,29-trihydroxy-27-norlanost-8-en-24-one
(3beta,17alpha,23S,24S)-17,23-Epoxy-3,24,29-trihydroxy-27-norlanost-8-en-15-one
Esculentic acid (Phytolacca)
(3beta,17alpha,23R)-17,23-Epoxy-3,29-dihydroxy-27-norlanost-8-ene-15,24-dione
12beta-Hydroxy-3-oxochol-4-en-24-oic Acid
9,11alpha-epoxy-6alpha-acetoxy-cholest-7-en-3beta,5alpha,19-triol
3-Oxo-7alpha-hydroxy-5alpha-cholestan-26-oic acid
Ganoderenic acid C
Ganoderic acid beta
Ganolucidic acid D
Ganoderic acid A
3beta,6beta-Dihydroxychol-4-en-24-oic Acid
Dihydrozeatin-9-N-glucoside-O-glucoside
4-(2-hydroxy-3-isopropyl-aminopropyl)benzoic scid
4-(2-Aminophenyl)-2,4-dioxobutanoic acid
Diferuloylputrescine
methyl 9,10-Epoxy-11,13-docosadiynoate
Thiamine
25-Hydroxyvitamin D3-26,23-lactol
1beta,3alpha,7alpha,12alpha-Tetrahydroxy-5beta-cholestan-26-oic acid
Fosfomycin
Oxoglutaric acid
Phosphoglycolic acid
Amoritin
Methionyl-Lysine
Integerressine
Cucurbitacin E
Isoamoritin
Pinnasterol
3a,7b,12b-Trihydroxy-5b-cholanoic acid
PE(20:3(5Z,8Z,11Z)/20:0)
Diadenosine tetraphosphate
(22E)-3beta-Hydroxy-5alpha-chola-7,22-dien-24-oic Acid
3alpha,12alpha,16alpha-Trihydroxy-5beta-cholan-24-oic Acid
PS(18:0/22:6(4Z,7Z,10Z,13Z,16Z,19Z))
1alpha-Hydroxy-3-oxo-5beta-cholan-24-oic Acid
5beta-Chola-3,8(14),11-trien-24-oic Acid
beta-muricholic acid
P1,P4-Bis(5'-adenosyl) tetraphosphate
I-Urobilin
3a,4b,12a-Trihydroxy-5b-cholanoic acid
Gsdmd-/-+NS
WT+NS
D
RichFactor
Number
P value
Primary bile acid biosynthesis
Sphingolipid metabolism
Sphingolipid signaling pathway
Leishmaniasis
Necroptosis
Regulation of lipolysis in adipocytes
Porphyrin and chlorophyll metabolism
Insulin resistance
Histidine metabolism
Aldosterone synthesis and secretion

Figure S2

Figure S2

Mice depleted with *Gsdmd* exhibited a distinct fecal metabolic profile after ConA treatment

A. Permutation test plot (200 cycles) between WT+NS and *Gsdmd*-/-+NS groups;

B. Permutation test plot (200 cycles) between WT+ConA and *Gsdmd*-/-+ConA groups;

C. Heatmap showing the top 50 most significantly differentially expressed fecal metabolites between WT+NS and *Gsdmd*-/-+NS groups;

D. The significant KEGG pathways enriched by the fecal differential metabolites of WT+NS and *Gsdmd*-/-+NS groups.

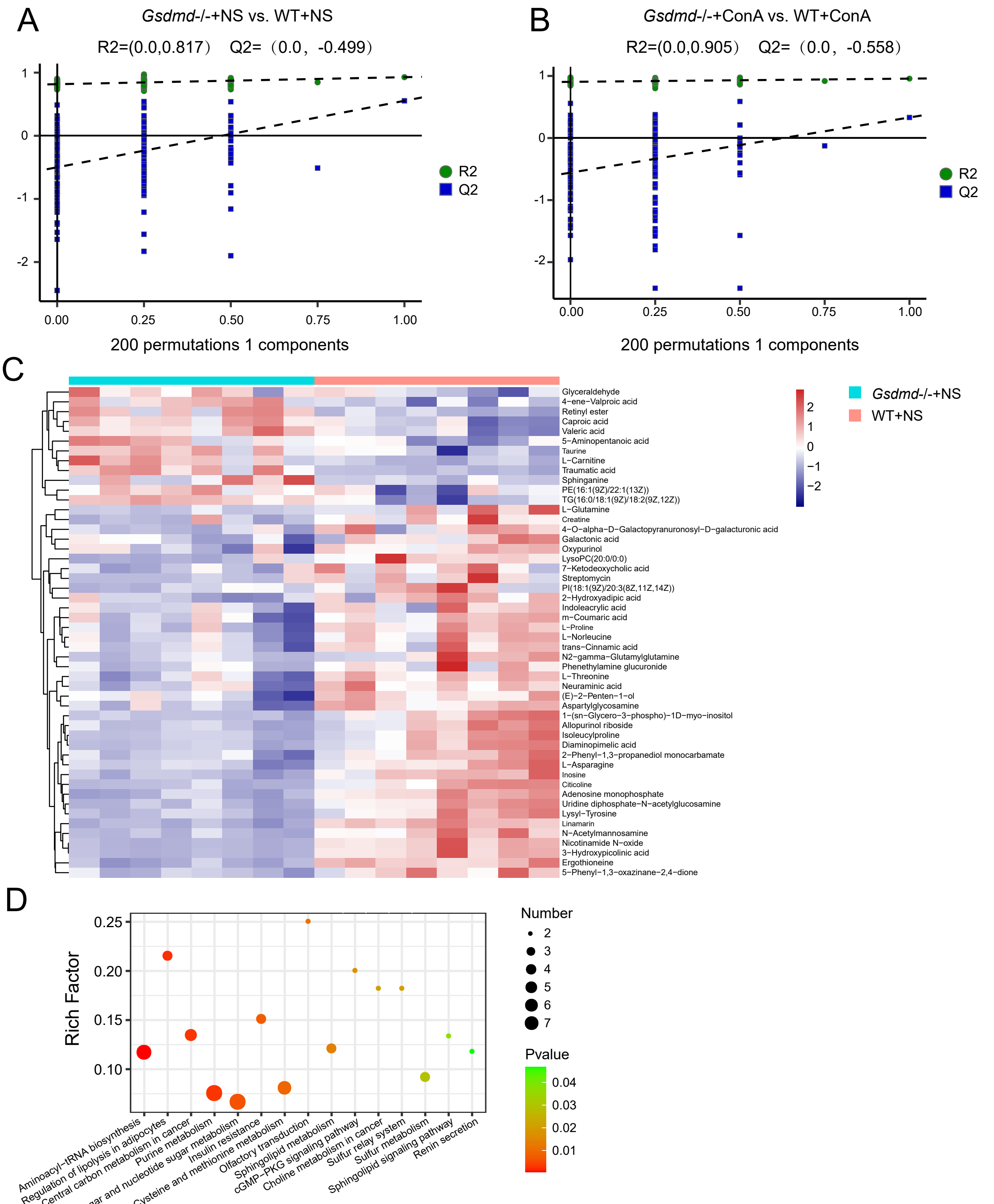
A
Gsdmd-/-+NS vs. WT+NS
R2=(0.0,0.817） Q2=（0.0，-0.499）
1
0
-1
-2
0.00
0.25
0.50
0.75
1.00
200 permutations 1 components
R2
Q2
B
Gsdmd-/-+ConA vs. WT+ConA
R2=(0.0,0.905） Q2=（0.0，-0.558）
1
0
-1
-2
0.00
0.25
0.50
0.75
1.00
200 permutations 1 components
R2
Q2
C
Glyceraldehyde
4−ene−Valproic acid
Retinyl ester
Caproic acid
Valeric acid
5−Aminopentanoic acid
Taurine
L−Carnitine
Traumatic acid
Sphinganine
PE(16:1(9Z)/22:1(13Z))
TG(16:0/18:1(9Z)/18:2(9Z,12Z))
L−Glutamine
Creatine
4−O−alpha−D−Galactopyranuronosyl−D−galacturonic acid
Galactonic acid
Oxypurinol
LysoPC(20:0/0:0)
7−Ketodeoxycholic acid
Streptomycin
PI(18:1(9Z)/20:3(8Z,11Z,14Z))
2−Hydroxyadipic acid
Indoleacrylic acid
m−Coumaric acid
L−Proline
L−Norleucine
trans−Cinnamic acid
N2−gamma−Glutamylglutamine
Phenethylamine glucuronide
L−Threonine
Neuraminic acid
(E)−2−Penten−1−ol
Aspartylglycosamine
1−(sn−Glycero−3−phospho)−1D−myo−inositol
Allopurinol riboside
Isoleucylproline
Diaminopimelic acid
2−Phenyl−1,3−propanediol monocarbamate
L−Asparagine
Inosine
Citicoline
Adenosine monophosphate
Uridine diphosphate−N−acetylglucosamine
Lysyl−Tyrosine
Linamarin
N−Acetylmannosamine
Nicotinamide N−oxide
3−Hydroxypicolinic acid
Ergothioneine
5−Phenyl−1,3−oxazinane−2,4−dione
2
1
0
−1
−2
Gsdmd-/-+NS
WT+NS
D
Rich Factor
0.25
0.20
0.15
0.10
Number
2
3
4
5
6
7
Pvalue
0.04
0.03
0.02
0.01
Aminoacyl−tRNA biosynthesis
Regulation of lipolysis in adipocytes
Central carbon metabolism in cancer
Purine metabolism
Amino sugar and nucleotide sugar metabolism
Insulin resistance
Cysteine and methionine metabolism
Olfactory transduction
Sphingolipid metabolism
cGMP−PKG signaling pathway
Choline metabolism in cancer
Sulfur relay system
Sulfur metabolism
Sphingolipid signaling pathway
Renin secretion

Figure S3

Figure S3

Gsdmd knockout changed the hepatic metabolic profile after ConA challenge

A. Permutation test plot (200 cycles) between WT+NS and *Gsdmd*-/-+NS groups;

B. Permutation test plot (200 cycles) between WT+ConA and *Gsdmd*-/-+ConA groups;

C. Heatmap showing the top 50 most significantly differentially expressed hepatic metabolites between WT+NS and *Gsdmd*-/-+NS groups;

D. The significant KEGG pathways enriched by the hepatic differential metabolites of WT+NS and *Gsdmd*-/-+NS groups.

Table S1

Liver function indexes	Concentration (U/L) in groups			
	WT+NS	WT+ConA	*Gsdmd*-/-+NS	*Gsdmd*-/-+ConA
ALT	43.34±2.984	3414±500.1***	38.98±3.861$^{###}$	15046±4355$^{\$}$
AST	155±13.4	2206±256.6***	145.4±12.12$^{###}$	10807±2826$^{\$}$

Data are shown as the mean ± SEM, *, P<0.05; **, P<0.01; ***, P<0.001, compared to WT+NS group; $^{\#}$, P<0.05; $^{\#\#}$, P<0.01; $^{\#\#\#}$, P<0.001, compared to *Gsdmd*-/-+ConA group; $^{\$}$, P<0.05; $^{\$\$}$, P<0.01; $^{\$\$\$}$, P<0.001, compared to WT+ConA group.

Table S2

Inflammatory cytokines	Cytokine concentration (pg/ml) in groups			
	WT+NS	WT+ConA	*Gsdmd*-/-+NS	*Gsdmd*-/-+ConA
Eotaxin	167.2±17.52	4638±481.3***	269.2±54.92##	8340±2190
G-CSF	721.2±238.3	102583±9329***	575±167.6##	135460±19193
GM-CSF	0.001±0	178.1±7.05***	0.001±0##	214.9±8.054$$
IFN-γ	4.298±0.9226	377.8±28.27**	4.924±1.702##	2789±585.7$$$
IL-1α	7.84±0.6929	105±7.605**	11.06±1.224##	127.6±9.307
IL-1β	5.855±0.7401	16.12±1.124**	3.475±0.825##	39.67±7.165$$
IL-2	1.618±0.2935	257.2±29.87***	1.033±0.4766##	111±9.441$$$
IL-3	2.695±0.4854	30.48±1.414***	2.27±0.6778##	39.55±2.496$$
IL-4	0.08733±0.06548	9.471±0.5438***	0.7908±0.7898##	13.06±2.667
IL-5	10.1±3.424	222.8±29.31**	18.17±8.866##	104.5±12.98$$
IL-6	53.88±29.62	3976±470.9***	11.83±4.312##	8437±2743
IL-9	0.001±0	92.29±4.737**	0.001±0##	148.5±21.49$$
IL-10	11.8±3.165	284.9±22.43***	21.16±5.291##	257.9±25.99
IL-12(p40)	1003±52.2	4806±364.6***	1048±167.6##	2856±267.8$$
IL-12(p70)	30.35±5.496	579.2±62.49***	11.41±8.006##	411.3±32.54$
IL-13	14.6±8.03	346.7±15.62**	46±10.81##	418.1±25.99$
IL-17A	40.2±7.513	88.77±9.632**	24.09±6.923##	203.3±30.53$$
KC	73.17±17.95	5615±614.1***	79.34±20.55##	13099±2357$$$
MCP-1	128.8±16.58	24949±2204***	338.9±104.3##	20810±2915
MIP-1α	1.995±0.1584	202.4±18.02**	2.544±0.199##	370.5±86.95
MIP-1β	14.9±9.592	3731±223.5***	31.07±9.566##	3900±487.8
RANTES	165.5±10.74	3506±273.8***	221.2±28.44##	4086±598
TNF-α	18.82±6.58	309.7±13.18**	10.19±2.695##	363.4±19.25$$

Data are shown as the mean ± SEM, *, P<0.05; **, P<0.01; ***, P<0.001, compared to WT+NS group; #, P<0.05; ##, P<0.01; ###, P<0.001, compared to *Gsdmd*-/-+ConA group; $, P<0.05; $$, P<0.01; $$$, P<0.001, compared to WT+ConA group.

Table S3

Comparison of the gut microbial composition of WT+NS, WT+ConA, *Gsdmd*-/-+NS, and *Gsdmd*-/-+ConA groups.

	PERMANOVA			Anosim	
	F-Model	R2	P-value	R-value	P-value
WT+ConA vs. WT+NS	3.0694	0.20368	0.001	0.3672	0.009
Gsdmd-/-+NS vs. WT+NS	10.632	0.49149	0.001	0.996	0.001
Gsdmd-/-+ConA vs. WT+ConA	7.5143	0.34927	0.001	0.9051	0.001
Gsdmd-/-+ConA vs. *Gsdmd*-/-+NS	2.6776	0.17079	0.001	0.2872	0.009

Table S5 Primer used for RT qPCR

Target gene	Forward primer 5’-3’	Reverse primer 5’-3’
Gapdh	TGCGACTTCAACAGCAACTC	ATGTAGGCAATGAGGTCCAC
Ifng	ACAGCAAGGCGAAAAAGGATG	TGGTGGACCACTCGGATGA
Tnf	CCCTCACACTCAGATCATCTTCT	GCTACGACGTGGGCTACAG
Il17a	TTTAACTCCCTTGGCGCAAA	CTTTTCCCTCCGCATTGACAC
Il2	TCTGCGGCATGTTCTGGATTT	ATGTGTTGTCAGAGCCCTTTAG
Il1b	GAAATGCCACCTTTTGACAGTG	CTGGATGCTCTCATCAGGACA
Il12a	CTGTGCCTTGGTAGCATCTATG	GCAGAGTCTCGCCATTATGATTC
Tlr4	ATGGCATGGCTTACACCACC	GAGGCCAATTTTGTCTCCACA
Cd14	ACTTCTCAGATCCGAAGCCAG	CCGCCGTACAATTCCACAT
Tjp1	GCCGCTAAGAGCACAGCAA	GCCCTCCTTTTAACACATCAGA
Ocln	TGAAAGTCCACCTCCTTACAGA	CCGGATAAAAAGAGTACGCTGG
Cldn4	ATGGCGTCTATGGGACTACAG	GAGCGCACAACTCAGGATG
Muc2	ATGCCCACCTCCTCAAAGAC	GTAGTTTCCGTTGGAACAGTGAA
Reg3g	GAAGCCAGATCCCGAAACCA	GAATCGGTAGACATCGCCGT
